# Three Isomeric Dioctyl Derivatives of 2,7-Dithienyl[1]benzo-thieno[3,2-b][1]benzothiophene: Synthesis, Optical, Thermal, and Semiconductor Properties

**DOI:** 10.3390/ma18040743

**Published:** 2025-02-07

**Authors:** Lev L. Levkov, Nikolay M. Surin, Oleg V. Borshchev, Yaroslava O. Titova, Nikita O. Dubinets, Evgeniya A. Svidchenko, Polina A. Shaposhnik, Askold A. Trul, Akmal Z. Umarov, Denis V. Anokhin, Martin Rosenthal, Dimitri A. Ivanov, Victor V. Ivanov, Sergey A. Ponomarenko

**Affiliations:** 1 Enikolopov Institute of Synthetic Polymeric Materials Russian Academy of Sciences, Profsoyuznaya Str. 70, Moscow 117393, Russia; l.levkov@ispm.ru (L.L.L.); surinnm@ispm.ru (N.M.S.); borshchev@ispm.ru (O.V.B.); yaroslava.titova@ispm.ru (Y.O.T.); n.dubinec@ispm.ru (N.O.D.); svidchenko@ispm.ru (E.A.S.); polinashaposhnik@ispm.ru (P.A.S.); trul@ispm.ru (A.A.T.); 2 National Research Centre «Kurchatov Institute», Novatorov Str. 7A-1, Moscow 119421, Russia; 3 Faculty of Chemistry, Lomonosov Moscow State University, Leninskie Gory 1/73, Moscow 119991, Russia; umarovakmalum@gmail.com (A.Z.U.); anokhin@icp.ac.ru (D.V.A.); dimitri.ivanov@uha.fr (D.A.I.); 4 Federal Research Center of Problems of Chemical Physics and Medicinal Chemistry RAS, Chernogolovka, Moscow 142432, Russia; 5 Faculty of Chemistry, KU Leuven, Celestijnenlaan 200F, P.O. Box 2404, B-3001 Leuven, Belgium; martin.rosenthal@esrf.fr; 6 Institut de Sciences des Matériaux de Mulhouse-IS2M, CNRS UMR 7361, Jean Starcky, 15, F-68057 Mulhouse, France; 7 Moscow Center for Advanced Studies, Kulakova Str. 20, Moscow 123592, Russia; ivanov.vv@mipt.ru

**Keywords:** organic semiconductor, solution processing, [1]benzothieno[3,2-b]benzothiophene, cross-coupling, organic field-effect transistor, in situ X-ray measurements

## Abstract

Organic semiconductor materials are interesting due to their application in various organic electronics devices. [1]benzothieno[3,2-b][1]benzothiophene (BTBT) is a widely used building block for the creation of such materials. In this work, three novel solution-processable regioisomeric derivatives of BTBT—2,7-bis(3-octylthiophene-2-yl)BTBT (**1**), 2,7-bis(4-octylthiophene-2-yl)BTBT (**2**), and 2,7-bis(5-octylthiophene-2-yl)BTBT (**3**)—were synthesized and investigated. Their optoelectronic properties were characterized experimentally by ultraviolet–visible and fluorescence spectroscopy, time-resolved fluorimetry, and cyclic voltammetry and studied theoretically by Time-Dependent Density Functional Theory calculations. Their thermal properties were investigated by a thermogravimetric analysis, differential scanning calorimetry, polarizing optical microscopy, and in situ small-/wide-angle X-ray scattering measurements. It was shown that the introduction of alkyl substituents at different positions (3, 4, or 5) of thiophene moieties attached to a BTBT fragment significantly influences the optoelectronic properties, thermal stability, and phase behavior of the materials. Thin films of each compound were obtained by drop-casting, spin-coating and doctor blade techniques and used as active layers for organic field-effect transistors. All the OFETs exhibited p-channel characteristics under ambient conditions, while compound **3** showed the best electrical performance with a charge carrier mobility up to 1.1 cm^2^·V^−1^s^−1^ and current on/off ratio above 10^7^.

## 1. Introduction

**Figure 1:**
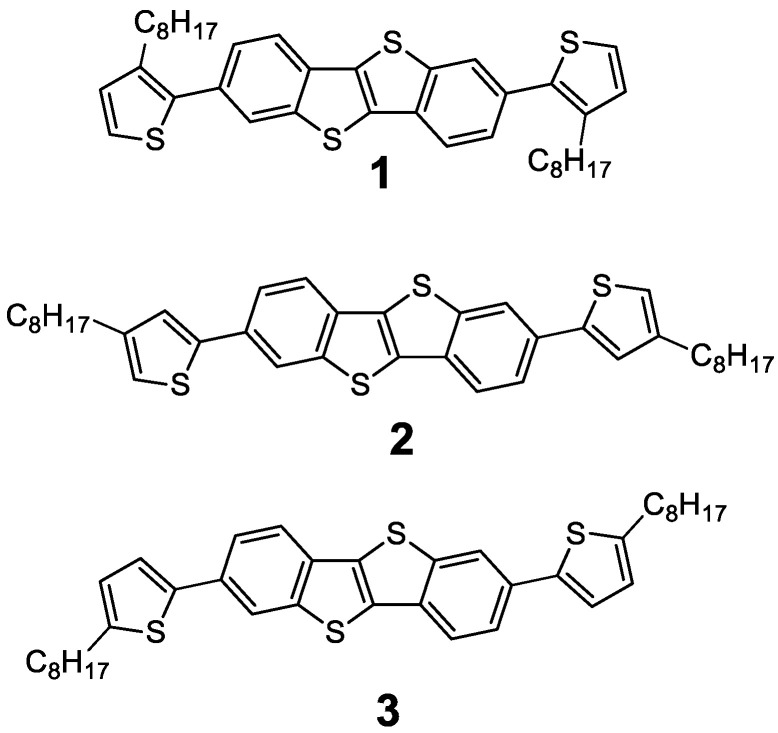
Chemical structures of novel BTBT derivatives.

Derivatives of [1]benzothieno[3,2-b][1]benzothiophene (BTBT) are one of the most popular types of organic semiconductors (OSCs) used in organic field-effect transistors (OFETs) [1]. The high chemical and thermal stability of the BTBT fragment allows variation in conjugated fragments and solubilizing groups or groups that contribute to the self-organization of semiconductor molecules in smectic mesophases [2] or self-assembled thin film monolayers and crystals [3,4]. The high degree of planarity of molecular semiconductors and the ability to self-organize [5] along with high processability allow the use of BTBT derivatives in various organic electronic devices with different configurations: OFETs for transistor arrays of high-performance devices [6,7] or gas sensors and an “electronic nose” based on them [8], electrolyte-gated organic field-effect transistors (EGOFETs) for chemical and biosensors in a water-based environment [9,10], organic light-emitting field-effect transistors [11], phototransistors [12], memristors [13], and organic and hybrid photovoltaics [14,15]. The BTBT molecule and its derivatives **1**, **2**, and **3** (Figure 1) belong to the class of heterocyclic compounds. The relationship between the absorption–fluorescence properties and the structure of alike heterocyclic compounds has been studied in detail both in classical works [16,17,18] and specifically for BTBT derivatives in recent works [19,20]. The disadvantage of alkyl derivatives of BTBT is usually the low stability of the active layer [21]. Phenyl-substituted derivatives of BTBT have rather low solubility and, as a result, devices with high mobility in most cases can be obtained only by the vacuum deposition of an organic semiconductor. One of the possible compromise solutions between intrinsic mobility and good manufacturability is the use of thiophenes as conjugated substituents of a BTBT core [22,23].

Herein, we introduce three newly synthesized regioisomeric compounds with n-octylthiophene substituents attached to 2,7 positions of a BTBT annulated core (Figure 1). Octyl chains were introduced in three possible positions of thiophene (2, 3, or 4) in order to analyze the effect of alkyl chain position on optoelectronics, thermo-oxidative stability, phase transitions, and electrophysical properties. Thermal, optical, and electrochemical properties of the BTBT-based compounds were characterized by a thermogravimetric analysis (TGA), differential scanning calorimetry (DSC), ultraviolet–visible (UV–Vis) absorption and fluorescence spectroscopy, time-resolved fluorimetry, and cyclic voltammetry (CV). Theoretical calculation based on Time-Dependent Density Functional Theory (TD DFT) was performed to evaluate frontier molecular orbital energy levels and to clarify experimental observations. Also, variable-temperature in situ small-/wide-angle X-ray scattering (SAXS/WAXS) measurements and polarized optical microscopy (POM) were used to verify phase transitions of each compound. The OFET fabrication, comprising solution processing of novel OSCs used in drop-casting, spin-coating, and doctor blade techniques, was carried out to analyze the electrical performance of each new compound.

## 2. Materials and Methods

### 2.1. Materials

Unless otherwise noted, reagents and solvents were purchased from commercial sources and used without further purification. 4,4,5,5-tetramethyl-2-(3-octylthiophen-2-yl)-1,3,2-dioxaborolane [24], 4,4,5,5-tetramethyl-2-(4-octylthiophen-2-yl)-1,3,2-dioxaborolane [25], 4,4,5,5-tetramethyl-2-(5-octylthiophen-2-yl)-1,3,2-dioxaborolane [26], [1]benzothieno[3,2-b][1]benzothiophene (BTBT) [27], 2,7-dioctyl[1]benzothieno[3,2-b][1]benzothiophene (C8-BTBT) [28], and 2,7-dibromo[1]benzothieno[3,2-b][1]benzothiophene [29] were synthesized according to previously reported methods. Freshly distilled toluene from calcium hydride was used for the reactions.

### 2.2. Synthesis of Organic Semiconductors

#### 2.2.1. Synthesis of 2,7-bis(3-Octylthiophen-2-yl)[1]benzothieno[3,2-b][1]benzothiophene (**1**)

2,7-dibromo[1]benzothieno[3,2-b][1]benzothiophene (0.80 g, 2.0 mmol) and 4,4,5,5-tetramethyl-2-(3-octylthiophen-2-yl)-1,3,2-dioxaborolane (1.42 g, 4.4 mmol) were placed in a 100 mL two-neck round-bottom flask. The flask was evacuated and purged with argon. After that, the cross-coupling catalyst Pd(PPh_3_)_4_ (93 mg, 0.1 mmol) was added and the flask was evacuated and purged with argon. In total, 25 mL of toluene, 4 mL of ethanol, and 4 mL of 2 M aqueous sodium carbonate solution were added through the septum. The mixture was refluxed for 6 h under an argon atmosphere. After cooling to room temperature, the bottom aqueous layer was taken out and the organic phase was filtered through a pad of silica gel. The solvent was removed under reduced pressure and the residue (0.90 g, 85% yield) was purified by column chromatography on silica gel (eluent—cyclohexane) and subsequent recrystallization using n-butanol afforded compound **1** as an off-white solid (0.65 g, 60% yield). ^1^H NMR (300 MHz, CDCl_3_) δ, ppm: 7.97 (dd, J = 1.6, 0.6 Hz, 2 H), 7.90 (dd, J = 8.3, 0.6 Hz, 2 H), 7.54 (dd, J = 8.2, 1.5 Hz, 2 H), 7.28 (d, J = 5.2 Hz, 2 H), 7.02 (d, J = 5.2 Hz, 2 H), 2.76–2.68 (m, 4 H), 1.70–1.58 (m, 4 H), 1.36–1.20 (m, 20 H), 0.90–0.82 (m, 6 H). ^13^C NMR (75 MHz, CDCl_3_) δ, ppm: 142.70, 139.17, 137.24, 133.65, 132.05, 131.94, 129.63, 126.64, 124.48, 124.07, 121.35, 31.84, 30.97, 29.46, 29.35, 29.28, 29.23, 22.64, 14.08.

ESI-MS (TOF) *m*/*z*: calculated for C_38_H_44_S_4_^+^ (M^+^) 629.2399, found: *m*/*z* (M^+^) 629.2371. Elemental composition calculated for C_38_H_44_S_4_: C (72.56%) H (7.05%) S (20.39%); found: C (72.08%) H (7.08%) S (19.22%).

#### 2.2.2. Synthesis of 2,7-bis(4-Octylthiophen-2-yl)[1]benzothieno[3,2-b][1]benzothiophene (**2**)

2,7-dibromo[1]benzothieno[3,2-b][1]benzothiophene (0.74 g, 1.9 mmol) and 4,4,5,5-tetramethyl-2-(4-octylthiophen-2-yl)-1,3,2-dioxaborolane (1.32 g, 4.1 mmol) were placed in a 100 mL two-neck round-bottom flask. The flask was evacuated and purged with argon. After that, the cross-coupling catalyst Pd(PPh_3_)_4_ (62 mg, 0.06 mmol) was added and the flask was evacuated and purged with argon. In total, 30 mL of toluene, 4 mL of ethanol, and 4 mL of 2 M aqueous sodium carbonate solution were added through the septum. The mixture was refluxed for 6 h under an argon atmosphere. After cooling to room temperature, the bottom aqueous phase was taken out and the organic phase was diluted with toluene, filtrated over a thin pad of silica gel. The solvent was removed under reduced pressure. The residue (0.80 g, 65% yield) was recrystallized twice in tetrahydrofuran (THF), dissolved in hot cyclohexane, and passed through a silica gel pad to afford compound **2** as an off-white solid (0.30 g, 25%). ^1^H NMR (300 MHz, CDCl_3_) δ, ppm: 8.10 (d, J = 1.6 Hz, 2 H), 7.81 (d, J = 8.3 Hz, 2 H), 7.67 (dd, J = 8.3, 1.6 Hz, 2 H), 7.24 (d, J = 1.4 Hz, 1 H), 6.91 (d, J = 1.4 Hz, 2 H), 2.68–2.62 (m, 4 H), 1.75–1.64 (m, 6 H), 1.44–1.27 (m, 20 H), 0.95–0.87 (m, 6 H). ^13^C NMR (75 MHz, CDCl_3_) δ, ppm: 144.62, 143.71, 143.35, 133.74, 132.32, 132.21, 125.06, 123.40, 121.70, 120.81, 119.94, 31.93, 30.74, 30.51, 29.47, 29.42, 29.28, 22.66, 13.97. ESI-MS (TOF) *m*/*z*: calculated for C_38_H_44_S_4_^+^ (M^+^) 629.2399, found: *m*/*z* (M^+^) 629.2384. Elemental composition calculated for C_38_H_44_S_4_: C (72.56%) H (7.05%) S (20.39%); found: C (72.69%) H (7.11%) S (20.20%).

#### 2.2.3. Synthesis of 2,7-bis(5-Octylthiophen-2-yl)[1]benzothieno[3,2-b][1]benzothiophene (**3**)

2,7-dibromo[1]benzothieno[3,2-b][1]benzothiophene (0.74 g, 1.9 mmol) and 4,4,5,5-tetramethyl-2-(5-octylthiophen-2-yl)-1,3,2-dioxaborolane (1.32 g, 4.1 mmol) were placed in a 100 mL two-neck round-bottom flask. The flask was evacuated and purged with argon. After that, the cross-coupling catalyst Pd(PPh_3_)_4_ (62 mg, 0.06 mmol) was added and the flask was evacuated and purged with argon. In total, 30 mL of toluene, 4 mL of ethanol, and 4 mL of 2 M aqueous sodium carbonate solution were added through the septum. The mixture was refluxed for 6 h under an argon atmosphere. A yellow precipitate was formed. After cooling to room temperature, it was filtered off, and washed with water and acetone (0.60 g, 50% yield). After that, it was recrystallized twice in THF, dissolved in hot cyclohexane, and passed through a silica gel pad to afford compound **3** as a yellow solid (0.40 g, 35% yield). ^1^H NMR (300 MHz, CDCl_3_) δ, ppm: 7.97 (dd, J = 1.6, 0.6 Hz, 2 H), 7.90 (dd, J = 8.3, 0.6 Hz, 2 H), 7.54 (dd, J = 8.2, 1.5 Hz, 2 H), 7.28 (d, J = 5.2 Hz, 2 H), 7.02 (d, J = 5.2 Hz, 2 H), 2.76–2.68 (m, 4 H), 1.70–1.58 (m, 4 H), 1.36–1.2 (m, 20 H), 0.90–0.82 (m, 6 H). ^13^C NMR (75 MHz, CDCl_3_) δ, ppm: 146.35, 143.37, 141.41, 133.61, 132.32, 132.11, 125.24, 123.25, 123.22, 121.68, 120.57, 31.91, 31.65, 30.40, 29.38, 29.23, 29.19, 22.65, 13.96. ESI-MS (TOF) *m*/*z*: calculated for C_38_H_44_S_4_^+^ (M^+^) 629.2399, found: *m*/*z* (M^+^) 629.2389. Elemental composition calculated for C_38_H_44_S_4_: C (72.56%) H (7.05%) S (20.39%); found: C (72.62%) H (7.15%) S (20.05%).

### 2.3. Methods

The progress of the reactions and the purity of the products were monitored by GPC on a Shimadzu instrument (Tokyo, Japan) with an SPD-M10AVvp diode matrix used as a detector; a Phenomenex column (Torrance, CA, USA), 7.8 × 300 mm, with a Phenogel sorbent (pore diameter: 500 Å); and a THF eluent, with a column temperature of 40 °C and flow rate of 1 mL/min. For purification by column chromatography, silica gel 60 Å “Merck” (Darmstadt, Germany) was used. ^1^H NMR spectra were recorded on a Bruker WP250 SY spectrometer (300 MHz, Karlsruhe, Germany), using the residual CHCl_3_ signal (δ = 7.27 ppm) as an internal standard. ^13^C NMR spectra were recorded on a Bruker Advance II 300 spectrometer (Karlsruhe, Germany) at an operating frequency of 75 MHz. In the case of ^1^H NMR spectroscopy, the analyzed compounds were taken in the form of 0.5% solutions in CDCl_3_. In the case of NMR ^13^C spectroscopy, the analyzed compounds were taken in the form of 1–3% solutions in CDCl_3_.

High-resolution mass spectra (HRMS) were recorded on a Maxis instrument (Bruker Daltonics, Bremen, Germany) using electrospray ionization (ESI). The measurements were performed in a positive ion mode (interface capillary voltage—4500 V); the mass range was from *m*/*z* 50 to *m*/*z* 1800 Da; external calibration included an Electrospray Calibrant Solution. The carbon and hydrogen elemental analyses were carried out by an express gravimetric analysis. The method is based on the pyrolytic combustion of a 3–5 mg sample of the substance in a platinum crucible in a quartz tube in an oxygen flow at 1000 °C in the presence of lead oxide. The sample combustion results in the quantitative formation of CO_2_ and H_2_O, which are absorbed outside the combustion tube by ascarite and anhydrone, respectively. The C and H contents were calculated based on the weight gain of the absorption apparatus. The settling titration using barium chloride was applied to analyze sulfur. The method error is 0.30–0.50%.

The thermogravimetric analysis was carried out in the dynamic mode in the range of 30–700 °C using a Mettler Toledo TG50 (Mississauga, ON, Canada) system equipped with an M3 microbalance, which allows measuring the mass of samples in the range of 1–150 mg with an accuracy of 1 μg. The heating rate was chosen to be 20 °C/min. DSC curves were obtained using a Mettler Toledo DSC30 system (Mississauga, ON, Canada) at a heating rate of 10 °C/min.

Spectrally pure solvent THF was used in optical studies. Absorption spectra of molecular solutions (10^−5^ mol/L) of compounds in THF and thin films were measured on a UV-2501PC spectrophotometer, Shimadzu. Luminescence spectra of molecular solutions and thin films of the studied compounds were measured on a spectrophotometer—spectrofluorometer FLUORAN-2 VNIIOFI (Moscow, Russia). Optical bandgaps were estimated from the edge of the corresponding absorption spectra. Quantum yield was determined by the method of measuring the fluorescence of optically diluted solutions (A < 0.1) using a 1,4-bis(5-phenyloxazol-2-yl)benzene ethanolic solution (quantum yield: 95%) as a standard [30]. Fluorescence decay kinetics of solutions was studied using a FluoTime 300 time-correlated photon-counting spectrofluorimeter (PicoQuant, Berlin, Germany). Fluorescence spectra were obtained by excitation at a wavelength λ_ex_ = 375 ± 2 nm (laser diode source: LDH 375) and λ_ex_ = 275 ± 2 nm (laser diode source: LDH 275). The signal was recorded near the maximum of the fluorescence spectra.

The electrochemical properties of the compounds were evaluated by CV. Cyclic voltammograms were recorded on an Elins PI-50-Pro-2 potentiostat (Moscow, Russia) in acetonitrile under a nitrogen atmosphere using tetrabutylammonium hexafluorophosphate (Bu_4_NPF_6_, 0.1 M) as a supporting electrolyte. The scan rate was 200 mV/s. Glassy carbon working and counter electrodes were used, and the reference electrode was 3.5 M Ag/AgCl. All the potentials were calibrated with the standard ferrocene/ferrocenium redox couple (Fe(Cp)_2_/Fe(Cp)_2_^+^) (E_1/2_ = +0.39 V measured under identical conditions). All measurements were taken in the thin films deposited onto the working electrode surface using concentrated solutions of the compounds. The HOMO ($E_{HOMO}^{CV}$) and LUMO ($E_{LUMO}^{CV}$) energy levels were calculated according to Equations (1) and (2), respectively [31,32]:

(1) $$E_{HOMO}^{CV}={-e(E}_{ox\;vs(Fc/{Fc}^{+})}^{onset}+4.8)$$

(2) $$E_{LUMO}^{CV}={-e(E}_{red\;vs(Fc/{Fc}^{+})}^{onset}+4.8)$$

where *e* is the elementary charge; $E_{ox\;vs(Fc/{Fc}^{+})}^{onset}$ and $E_{red\;vs(Fc/{Fc}^{+})}^{onset}$ are the oxidation and reduction potentials, respectively.

All polarized optical images were obtained by using a polarizing microscope (Axioscop 40 A Pol, Zeiss AG, Oberkochen, Germany) and heating stage (THMS600, Linkam Scientific Instruments Ltd., Salfords, UK).

SAXS and WAXS experiments were conducted at the BM26 beamline of the European Synchrotron Radiation Facility (ESRF) in Grenoble, France. Two-dimensional X-ray scattering patterns were recorded using Pilatus 1 M and Pilatus 300 k (Dectris AG, Baden-Daettwil, Switzerland) detectors during heating (10 °C/min from room temperature to 350 °C) with a Linkam DSC600 heating stage (Linkam Scientific Instruments Ltd., Salfords, UK). Silver behenate and LaB_6_ were used to calibrate the norm of the reciprocal-space vector q.

### 2.4. Theoretical Calculations

Quantum-chemical DFT and TD DFT calculations were performed for each molecule investigated. The geometry of isolated molecules was optimized in the ground state using the DFT calculations with exchange-correlation functional PBE0 [33] and Ahlrich’s SVp basis set [34] and including D3BJ dispersion correction [35]. Full energies, HOMO/LUMO energies, and S_0_ → S_n_ energies were refined using the def2-TZVp basis set [36]. Flexible scanning for the dihedral angle between thiophene and BTBT moieties in compound **3** was made in order to determine changes in energy of S_0_ → S_1_ transition. All quantum-chemical calculations were conducted in the ORCA5 program [37].

### 2.5. OFET Fabrication and Characterization

OFETs were prepared by three different methods: spin coating (SC), a doctor blade (DB), and drop casting (DC) on silicon/SiO_2_ substrates in the bottom-gate bottom-contact (BC) or bottom-gate top-contact (TC) architecture. Highly doped silicon wafers with thermally grown 300 nm thick silica dioxide layers were used as the substrates. Source and drain electrodes (Au: 35 nm) with a channel length of 30 μm and a channel width of 1000 μm (a geometrical ratio W/L = 33) were thermally evaporated through a shadow mask either before (for BC) or after (for TC) the organic semiconducting (OSC) layer deposition.

In the case of bottom contacts, source and drain electrodes were subsequently treated with plasma using a low-pressure plasma system (Diener Electronic GmbH, Ebhausen, Germany) for 5 s and then modified by immersing the device for 15 min in a 15 mM pentafluorobenzenethiol (PFBT) solution in isopropanol and then placed in isopropanol for 5 min to remove the excess of PFBT.

In the case of the doctor blade deposition of OSC, the height of the blade above the table was 650 microns and the volume of the applied solution was 50 µL, blade speed was 0.1 or 0.3 mm/s, concentration of the OSC was varied between 2 and 15 g/L in toluene or dichlorobenzene (DCB), and table temperature was 30 or 85 °C for toluene and 85 or 140 °C for DCB solutions. In the case of spin coating, the following conditions were used: a rotation speed of 2000 rpm, toluene as a solvent, the volume of the applied solution was 100 µL, and the concentration of the OSC was 2 g/L or 3.67 g/L for its mixture with 14.68 g/L of polystyrene (PS). In the case of drop casting, 10 or 60 µL of 0.1 g/L solutions in DCB was applied.

To avoid current leaks, as well as to correctly determine the length (L) and width (W) of the channel, the perimeter of the device active area was carefully scratched. Electrical measurements were performed in air with a source meter (Keithley 2634B, Tektronix, Inc, Beaverton, OR, USA) using a probe station (ProbeStation 100, Printeltech, Moscow, Russia). The charge carrier mobility and threshold voltages were evaluated according to Shockley’s model [11] in a saturated regime according to Equation (3):

(3) $$I_{d}=\frac{W}{2L}\mu_{sat}C_{i}\left(V_{g}-V_{th}\right)$$

where $\mu_{sat}$ is the charge carrier mobility in the saturated regime, W is the channel width, L is the channel length, $V_{g}$ is the gate voltage, $V_{th}$ is the threshold voltage, and $C_{i}$ =11.5 nF cm^−2^ is the dielectric capacitance calculated from the thickness of the SiO_2_ layer (300 nm) and its dielectric constant (3.9).

## 3. Results and Discussion

### 3.1. Synthesis

**Figure 2:**
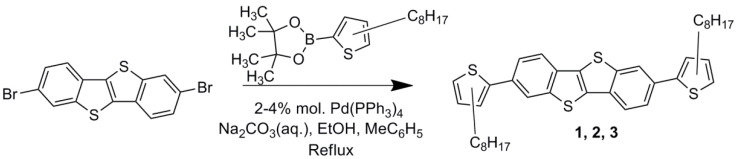
Synthesis scheme of compounds **1**, **2**, and **3**.

The synthesis of new OSCs was carried out under the conditions of the Suzuki cross-coupling reaction between 2,7-dibromo[1]benzothieno[3,2-b][1]benzothiophene and excess of boronic acid pinacol esters according to the reaction scheme in Figure 2. The position of alkyl substituents in the regioisomers was determined by their position in the corresponding initial n-octylthiophene pinacol esters: 3, 4, or 5. In all three cases, the reactions were fairly rapid (about 6 h) and no starting dibromide was detected in the reaction mixtures prior to the product isolation. Moderate yields of products **1**, **2**, and **3** (60%, 25%, and 35%, respectively) are associated with losses at the purification stages using chromatography and recrystallization. The chemical structure of the compounds obtained was approved by ^1^H and ^13^C NMR spectroscopy (Figures S1–S6). In the ^1^H NMR spectra of all three novel compounds, two doublets and a doublet of doublets of the same integral intensity in the region of 8.1–7.5 ppm are presented, indicating six protons of benzene rings of BTBT, while two doublets of the same integral intensity (compound **1**) or a doublet and a doublet of triplets (compounds **2** and **3**) in the region of 7.3–6.7 ppm correspond to four protons of double substitution in differently positioned thienyl fragments. There is a series of characteristic multiplets in the strong field corresponding to 34 protons of two octyl substituents. A detailed correlation between each H and C atom with a corresponding chemical shift in the ^1^H NMR spectra is given in Figures S1, S3 and S5 in the Supplementary Materials. In the ^13^C NMR spectra of these compounds, 11 signals in the region of 150–120 ppm and 8 signals in the region of 35–10 ppm are detected. It correlates well with the quantity of chemically equivalent carbon atoms in the aromatic BTBT and thiophene fragments as well as aliphatic substituents, respectively. The experimental masses of molecular ions of compounds 1–3 (Figures S7–S9) and the elemental composition correspond well to the calculated values. The purity of compounds **1**–**3** was >99% according to GPC analysis data (Figures S10–S12).

### 3.2. Photophysical Characterization and Theoretical Calculations

**Figure 3:**
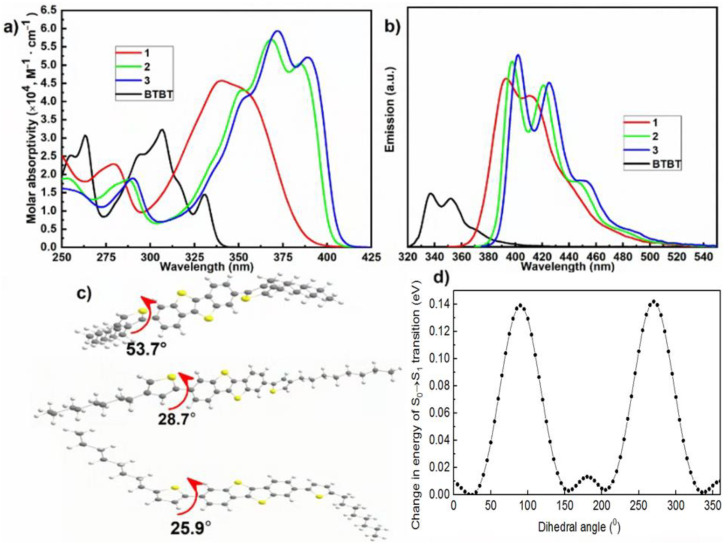
UV-vis absorption (**a**) and photoluminescence (**b**) spectra. Luminescence spectra were obtained by excitation at correspondence to main maxima wavelengths. Optimized ground state structures of studied regioisomers (**c**); change in energy of S0 → S1 transition against dihedral angle between thiophene and BTBT moieties in compound **3** (**d**).

UV–vis absorption and fluorescence spectroscopy, time-resolved fluorimetry, CV, and TD DFT calculations were employed to evaluate the effect of the position of alkyl substituents in thiophene rings on optoelectronic properties and figure out the HOMO/LUMO and bandgap (E_g_) energy levels of each compound. The absorption (molar absorptivity) and fluorescence spectra of diluted solutions of compounds **1**–**3** in THF are shown in Figure 3a,b; photophysical properties are highlighted in Table 1. Data for [1]benzothieno[3,2-b][1]benzothiophene (**BTBT**) obtained in the same conditions are provided for comparison.

In heterocyclic compounds, an unshared pair of heteroatom electrons is already involved in conjugation with the π electrons of the molecule in the ground state. Such compounds have a filled π orbital, the energy of which is greater than the energy of the upper filled π orbital of an unsubstituted hydrocarbon. In addition to the bands caused by π → π* transitions from this orbital, the absorption spectra of heterocyclic compounds contain bands caused by n → π* transitions. The presence of n → π* junctions with energy less than the energy of the longest-wavelength π → π* junction often leads to the suppression of π* → π fluorescence. The absorption spectrum of BTBT in the wavelength range of 250–350 nm has three bands (Figure 3a). The main contribution to the formation of these bands is made by three electron–vibrational transitions: S0 → S1, S0 → S2, and S0 → S6 (see Table S1 in the Supplementary Materials). The oscillator forces corresponding to purely electronic transitions are 0.14, 0.33, and 0.30. It is known that the values of the oscillator forces of the n → π* junctions do not exceed 10^−3^–10^−2^; therefore, the main absorption bands of BTBT are caused by π → π* junctions. The BTBT fluorescence spectrum in THF is shown in Figure 3b. It has a well-defined vibrational structure. Maximum fluorescence has a wavelength of 337 nm. The magnitude of the Stokes shift is rather small—540 cm^−1^. Thus, the observed fluorescence refers to the S1→S0 transition and it is π* → π fluorescence. The quantum yield of BTBT fluorescence in THF is 3 ± 1%, and the fluorescence lifetime is 0.19 ns. The fluorescence rate constant is 0.16 ns^−1^, and the singlet–triplet conversion rate constant is 5.1 ns^−1^ (Table 1). Such a high singlet–triplet conversion rate constant may be due to the presence of nπ*-type triplet states, whose energy is close to the energy of the S1S0 state. As it is known, the probability of a singlet–triplet conversion between the Sππ* and Tnπ* states is higher than those between the Sππ* and Tnπ* states [38]. The addition of conjugated electron-donor fragments to the heterocycle leads to the appearance of a new, longer-wavelength π,π* band. Indeed, after the addition of thiophene fragments to BTBT, intense long-wavelength bands appeared in the absorption spectra of compounds **1**, **2**, and **3** with peaks at wavelengths of 340, 385, and 389 nm, respectively. The absorption spectra of compounds **1**, **2**, and **3** contain intense long-wavelength bands with peaks at 340, 385, and 389 nm, respectively. While the spectra of compounds **2** and **3** have a pronounced vibrational structure, only one structureless band in the spectrum of 1 is observed (Figure 3a). The calculated values of the oscillator strength of purely electronic transitions corresponding to the long-wavelength absorption bands of compounds **1**, **2**, and **3** are 1.03, 1.46, and 1.58, respectively (Table S1 in the Supplementary Materials). The bathochromic shift relative to the maximum of the long-wavelength absorption band of BTBT is **1**—800 cm^−1^ (0.1 eV); **2**—4200 cm^−1^ (0.53 eV); and **3**—4500 cm^−1^ (0.56 eV). The values of the displacements of the long-wavelength bands of compounds **1**, **2**, and **3** correlate with the values of the torsion angles between BTBT and thiophene fragments: **1**—53.7°; **2**—28.7°; and **3**—25.9°. For direct comparison, calculations were also made for 2,7-bis(thiophene-2-yl)BTBT (BTBT-T), which has two thiophene rings attached to a central BTBT fragment, but without any alkyl chains (Table S1 in the Supplementary Materials). It can be clearly seen that a BTBT-T molecule has the torsion angles of 25.9°, which is very similar to those for compound **3**. The other calculated parameters for a BTBT-T molecule are also very close to those for compound **3**, which makes these molecules very similar by their properties. Optimized ground state structures of the regioisomers investigated are provided in Figure 3c. A decrease in the torsion angle leads to an increase in the degree of π conjugation and, consequently, to a decrease in the transition energy S0 → S1. The graph of the dependence of the change in the energy of the long-wave transition on the change in the angle of rotation is shown in Figure 3d.

The fluorescence spectra of compounds **1**, **2**, and **3** are shown in Figure 3b. Analogously to the absorption spectra, the vibrational structure in the fluorescence spectra of compounds **2** and **3** is clearly expressed; the vibrational structure of the spectrum of compound **1** is observed to a much lesser extent. The reason for the different structure of the absorption and fluorescence spectra of compounds **1**, **2**, and **3** is related to the different values of the torsion angles. The change in S0–S1 transition energy is 0.018 eV by a ±5° declination of the thiophene moiety. So, that is for 53.7° (**1**); 0.003 eV for 28.7° (**2**); and 0.002 eV for 25.9° (**3**). Consequently, rotational vibrations of thiophene fragments due to interaction with solvent molecules lead to a greater broadening of the spectra of compounds 1 than of compounds **2** and **3**. The Stokes shift for compound **1** is 2200 cm^−1^; for compound **2**, it is 640 cm^−1^; and for compound **3**, it is 630 cm^−1^. The magnitude of the Stokes shift also correlates with the magnitude of the torsion angles between BTBT and thiophene fragments. The larger the torsion angle, the greater the energy loss due to rotational vibrations of the thiophene fragments and, consequently, the greater the Stokes shift.

The quantum yield and lifetime of the fluorescence increase with an increasing degree of π conjugation are as follows: 40% and 0.52 ns (**1**), 63% and 0.78 ns (**2**), and 73% and 0.82 ns (**3**). When going from **1** to **3**, the fluorescence rate constant increases by 16%, while the singlet–triplet conversion rate constant decreases by 4 times (Table 1). Therefore, the increase in the quantum yield of fluorescence is mainly due to a decrease in the probability of singlet–triplet conversion.

It was noted above that the high rate of singlet–triplet conversion in BTBT is due to the presence of nπ*-type triplet states, whose energy is close to the energy of π,π*-state S1S0. The addition of conjugated thiophene fragments led to the appearance of intense long-wavelength π,π* absorption bands in compounds **1**, **2**, and **3**. It is known that singlet and triplet bands of the nπ* type shift to the short-wavelength region of the spectrum as the degree of conjugation increases [18]. The greater the degree of the conjugation of thiophene fragments with BTBT, the higher the nπ*-type triplet states turn out to be in relation to the π,π*-state S1S0. This reduces the probability of a singlet–triplet conversion in compounds **1**, **2**, and **3**. The long-wavelength shift in the π,π* absorption bands in compounds **1**, **2**, and **3** also leads to a decrease in the splitting energy between S1 and T1 π,π* states; however, the energy difference remains quite large (10,400 cm^−1^ for compound **3**; see Table S1 in the Supplementary Materials) and the probability of a singlet–triplet conversion between the Sππ* states and Tnπ* does not increase.

### 3.3. Bandgap and Frontier Molecular Orbital Energy Value Estimation

Frontier molecular orbital energy values were estimated both theoretically by DFT calculations and experimentally by CV measurements. Bandgaps were additionally calculated from the absorption measurements in solutions and polycrystalline thin films (see Figure S14 in the Supplementary Materials). It should be noted that long-wavelength absorption spectra of the thin films coincide well with their excitation spectra that allowed, with a good precision, the calculation of their bandgaps (Table 2).

**Figure 4:**
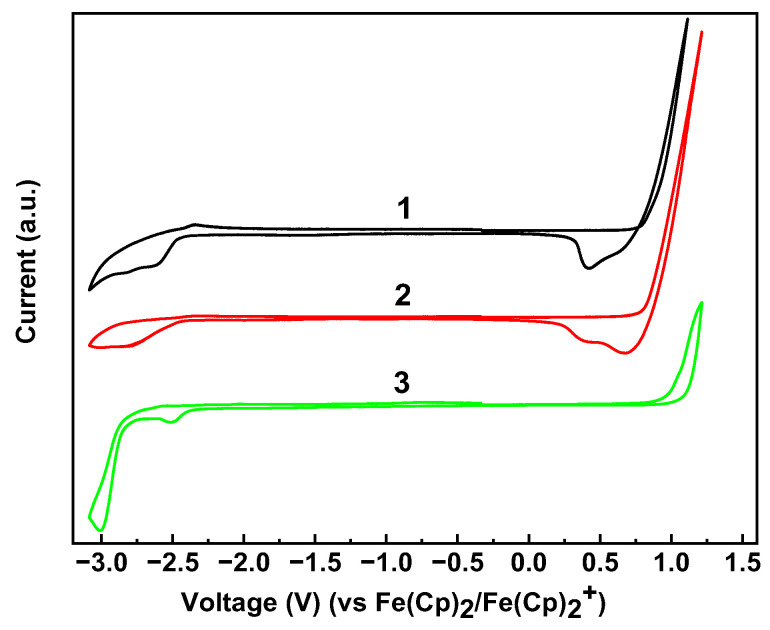
Cyclic voltammetry curves of thin films of compounds **1**–**3** deposited on glassy carbon electrode.

In CV, every compound was deposited on a glassy carbon electrode from a toluene solution and analyzed at room temperature using ferrocene/ferrocenium as a reference (Figure 4). The potentials of oxidation/reduction processes of compounds **1**, **2**, and **3** were observed at 0.79/−2.45, 0.81/−2.45, and 0.97/−2.37 V, respectively (Figure 4). The corresponding HOMO and LUMO energy levels were calculated by Equations (1) and (2). DFT calculations performed for individual molecules showed that HOMO energy values are in rather good agreement with the CV experimental data, but the LUMO values are 0.5–0.65 eV higher than those obtained experimentally. This causes the bandgap data to diverge. The comparison of the experimental and calculated frontier orbital energies and bandgaps is provided in Table 2. There is much better agreement between the bandgap values obtained experimentally from CV and absorption spectra measurements. As expected, bandgaps estimated from thin film measurements are 0.08–0.12 eV smaller than those obtained from solution measurements. It is well known that the aggregation of conjugated molecules in thin films influence their absorption spectra and bandgaps. Absorption measurements both from a solution and thin films show a clear tendency of the bandgap decreasing when going from compound **1** to compound **3**, like how it was calculated by DFT. This is not the case for CV measurements, which could be related to low quality of the films of compound **3** measured due to its low solubility.

### 3.4. Thermal Properties

The characterization of thermal and thermal-oxidative stability, as well as the regularities of phase transitions of OSCs, is an integral part of the development of an effective method for manufacturing electronic and optoelectronic devices. Thermal properties of the compounds synthesized were characterized by TGA and DSC measurements.

All three compounds studied have a fairly high stability in the inert atmosphere: their temperatures of 5% weight loss were 378, 429, and 427 °C of compounds **1**, **2**, and **3**, respectively (see Figure S15 in the Supplementary Materials). However, their thermal-oxidative stability in the air was significantly reduced to 350, 276, and 257 °C, respectively. Therefore, in the argon, all the compounds can be heated to the temperatures corresponding to all their transitions. Thermal stability increases when going from compound **1** to compound **3**. In the air, the opposite trend is observed. The decomposition of compound **2** begins upon transition to an isotropic liquid; compound **3** cannot be melted in the presence of oxygen. Compound **1** is stable during multiple remelting cycles in air.

**Figure 5:**
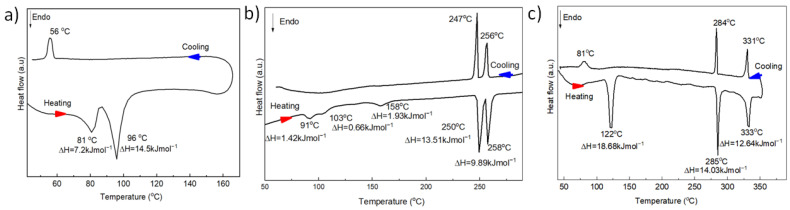
The first heating and cooling DSC curves of compounds **1**–**3** (in argon atmosphere): (**a**)—compound **1**, (**b**)—compound **2**, (**c**)—compound **3**.

To exclude any oxidation, DSC measurements were made under an argon atmosphere. The first heating and cooling DSC curves are shown in Figure 5, while data of the temperature, enthalpy, and entropy of the first-order phase transitions from the first heating DSC scans are summarized in Table 3. As can be seen, compound **1** shows two rather low-temperature phase transitions—at 81 and 96 °C—with corresponding enthalpies of 7.2 and 14.5 kJ/mol and entropies of 20.3 and 39.4 J mol^−^^1^K^−^^1^. Polarizing optical microscopy (POM) showed that the second phase transition is melting of the crystal phase into the isotropic liquid, which crystallizes under cooling (see Figure S16a in the Supplementary Materials). Thus, the first phase transition should be a crystal-to-crystal polymorphic phase transition.

The DSC heating scan of compound **2** shows five endothermic peaks corresponding to the first-order phase transitions: at 91, 103, 158, 250, and 258 °C. However, under cooling, only two exothermic peaks at 257 and 247 °C can be observed (Figure 5b), indicating that only two upper phase transitions are enantiotropic. Both of them also have the highest melting enthalpies and entropies—13.51 and 9.89 kJ mol^−^^1^ and 25.8 and 18.6 J mol^−^^1^K^−^^1^, respectively (at heating). POM investigations in cross-polarizers show that upper phase transition corresponds to isotropic melting, while cooling from the isotropic melt leads to the formation of poorly developed fan-like texture, which under further cooling transforms into the other fan-like structure (Figure S16b,c in the Supplementary Materials). Thus, two upper phases are liquid crystalline phases, the nature of which was clarified by SAXS/WASX measurements (see below).

Compound **3** shows three enantiotropic phase transitions at 122, 285, and 333 °C with rather high enthalpies of 18.68, 14.03, and 12.64 kJ mol^−^^1^, respectively, and corresponding entropies of 47.3, 25.1, and 20.9 6 J mol^−^^1^K^−^^1^ (at heating). POM investigations in cross-polarizers show that upper phase transition corresponds to isotropic melting, while cooling from the isotropic melt leads to the formation of poorly developed fan-like texture, which under further cooling somewhat changes (Figure S16d,e in the Supplementary Materials). The presence of fin-like structures indicates a liquid crystalline nature of two upper phases, the identification of which was made on the basis of in situ X-ray scattering (see below).

### 3.5. In Situ Synchrotron X-Ray Scattering

**Figure 6:**
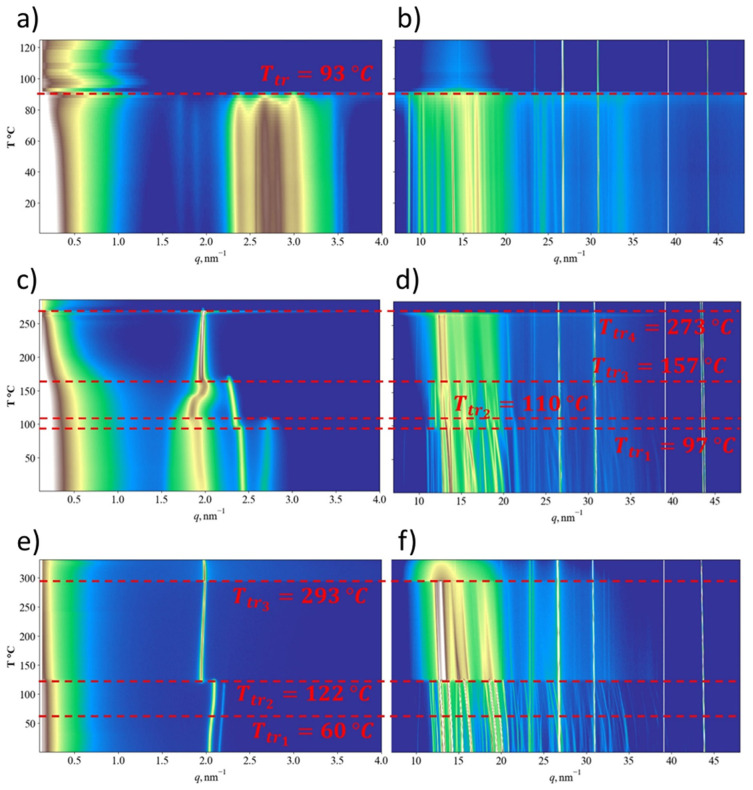
SAXS (left column) and WAXS (right column) profiles recorded during heating at 10 °C/min for the studied samples: compound **1** (**a**,**b**), compound **2** (**c**,**d**), and compound **3** (**e**,**f**). Red dashed lines mark the temperatures associated with phase transitions.

The complex thermal behavior of BTBT derivatives and the direct influence of their phase structure on the performance of OPV devices and OFETs, where these molecules serve as active layers, highlight the necessity of employing in situ synchrotron X-ray scattering for comprehensive characterization [39,40]. In the present work, the structure and phase transitions of compounds **1**, **2**, and **3** were analyzed with variable-temperature synchrotron small- and wide-angle X-ray scattering. The results obtained during heating of the samples from room temperature at a rate of 10 K/s are shown in Figure 6.

At room temperature, compound **1** exhibits a diffraction pattern characteristic of a crystalline phase (Figure 6a,b). In the SAXS region, several sharp peaks appear within the q-range of 1.5 to 2.5 nm^−1^, with d-spacings approximately corresponding to the molecular length. The presence of multiple peaks in this range indicates a large unit cell volume, likely due to the non-extended conformation of compound **1**. At 93 °C, a direct transition from the crystalline phase to the isotropic state is observed, consistent with the DSC data. The absence of intermediate smectic phases may be attributed to the significant structural differences between the molecular arrangement in the crystalline unit cell and a smectic state. Notably, the first melting event detected in the DSC trace at 81 °C was not resolved in the SAXS/WAXS heating profiles and may be associated with the reorganization of small crystallites. The peaks observed in the WAXS region above the melting transition are attributed to reflections from the sample pan.

The thermal behavior of compound **2** exhibits significantly greater complexity compared to compound **1** (Figure 6c,d). At room temperature, the SAXS region displays three distinct peaks, while the WAXS region features multiple peaks, characteristic of a crystalline phase or a mixture of crystalline phases. Importantly, the SAXS peaks are not equidistant in q-space, indicating that the structure cannot be described as a simple layered arrangement. Upon heating, the first transition occurs in the range of 97–110 °C, marked by a stepwise shift in the SAXS peak at q = 2.4 nm^−1^, the disappearance of the peak at q = 2.7 nm^−1^, and a reorganization of crystalline WAXS peaks indicative of a crystal-to-crystal transition. The presence of two non-equidistant SAXS reflections up to 157 °C suggests the persistence of a three-dimensional crystal phase, beyond which the system transitions to a smectic B (SmB) phase. The SmB phase is characterized by hexagonal packing of rotationally disordered molecules in a two-dimensional lattice [41]. The DSC trace indicates that another transition may occur between the SmB and the isotropic phase, potentially forming a smectic A (SmA) phase characterized by positional disorder within the layers. However, due to the narrow temperature window of this transition, the temperature resolution of the synchrotron experiment may not have been sufficient to directly observe this intermediate phase.

**Figure 7:**
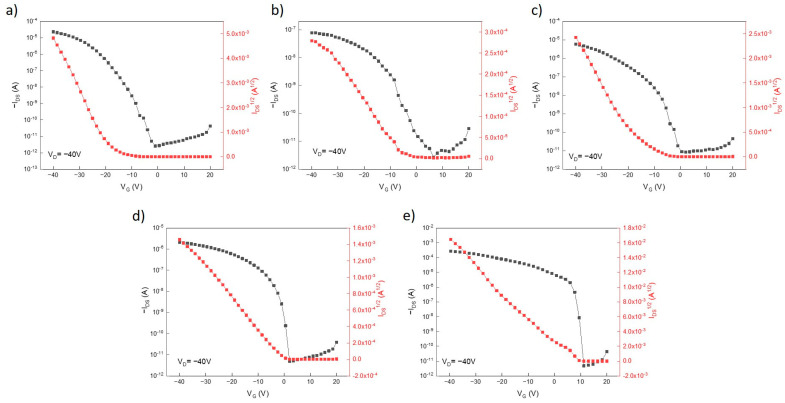
Transfer characteristics for the best devices in logarithmic (black) and square root (red) representations for compound **1** (**a**), compound **2** (**b**), and compound **3** ((**c**)—doctor blade/top contacts, (**d**)—doctor blade/bottom contacts, (**e**)—drop casting).

Compound **3** exhibits a double peak at q = 2.0–2.2 nm^−1^, characteristic of a non-layered crystal structure (Figure 7e). The first transition, observed at 60 °C in the WAXS profiles, is not clearly detected in the DSC trace and is likely associated with minimal structural changes during a crystal-to-crystal transition (Figure 6f). In contrast, the second transition at 122 °C results in the disappearance of the majority of WAXS reflections and indicates a transformation from the crystalline phase to the SmB phase. At 293 °C, all narrow WAXS reflections vanish, revealing the formation of an SmA structure. The observed structural transitions are summarized in Table 4.

### 3.6. OFET Device Characterization

Due to different solubility and phase behavior of compounds **1**–**3**, OFETs were prepared by three different solution-processing techniques, spin coating (SC), a doctor blade (DB), and drop casting (DC), using either bottom- or top-contact configurations. Varying conditions, concentrations of OSC, solvents, and application temperature allowed us to find better conditions for each of the compounds investigated. Characteristics of the best devices prepared from compounds **1**–**3** prepared by different methods are presented in Figure 7 and summarized in Table 5. For comparison reasons, the same devices were also prepared for a widely used organic semiconductor, **C8-BTBT**. As can be seen, methods and conditions for the best devices are different for each of the compounds investigated. Characteristics of the OFET from the other compounds under similar conditions can be found in the Supplementary Materials. Since there were clear nonlinearities in the OFET characteristics, effective mobilities μ eff. were estimated from reliability factor $r_{sat}$ introduced by V. Podzorov et al. [42]:

(4) $$r_{sat}=\frac{{\left(\frac{\sqrt{{\left|I_{sd}\right|}^{max\;}}-\sqrt{\left|I_{sd}^{0}\right|}}{{\left|V_{g}\right|}^{max}}\right)}^{2}}{\left(\frac{WC_{i}}{2L}\mu_{sat}\right)}=\frac{{\left(\frac{\sqrt{{\left|I_{sd}\right|}^{max\;}}-\sqrt{\left|I_{sd}^{0}\right|}}{{\left|V_{g}\right|}^{max}}\right)}^{2}}{{\left(\frac{\partial \sqrt{\left|I_{sd}\right|}}{\partial V_{g}}\right)}^{2}}$$

(5) $$\mu_{eff}=r\times \mu_{sat}$$

where $\mu_{sat}$ is the experimentally determined mobility; *L*, *W*, and $C_{i}$ are device parameters; and ${\left|I_{sd}\right|}^{max\;}$ is the experimental maximum source-drain current reached at the maximum gate voltage. $I_{sd}^{0}$ is the source-drain current at $V_{g}$ = 0.

As can be seen from the data presented, the effective field-effect mobility in the OFETs increases in the row of compounds **2** < **1** < **3**. For compound **1**, the best *μ_eff._* = 0.08 and an I_on/off_ ratio almost reaching 10^7^ was measured for OFET devices obtained by the spin-coating method with top contacts. For compound **2**, the best *μ_eff._* = 2.5 × 10^−4^ and an *I_on/off_* = 2.9 × 10^4^ was measured for OFET devices obtained by the doctor blade method with top contacts. For compound **3**, *μ_eff_.* increases from DB/BC to DB/TC devices and its maximal value was obtained for the drop-casting device, for which it reaches the value of 1.1 cm^2^V^−1^s^−1^ and the *I_on/off_* ratio exceeds 10^7^.

## 4. Conclusions

In this work, a series of three regioisomeric compounds with 3-, 4-, or 5-octylthiophene substituents attached to 2,7 positions of a BTBT annulated core was synthesized; their chemical structure and purity were proven by a combination of ^1^H and ^13^C NMR spectroscopy, HRMS, an elemental analysis, and GPC chromatography on an oligomeric column with a diode array detector.

The effect of the position of alkyl substituents in thiophene rings on optoelectronic properties of these compounds was investigated both experimentally and theoretically by UV–vis absorption and fluorescence spectroscopy, time-resolved fluorimetry, CV, and TD DFT calculation, which allowed us to evaluate the HOMO/LUMO and bandgap (*E_g_*) energy levels of each compound.

The in situ structural characterization of three regioisomeric compounds with n-octylthiophene substituents attached to the 2,7 positions of BTBT revealed complex phase transitions upon heating. Interestingly, at room temperature, all three compounds exhibit a bulky unit cell, distinct from the layered structures typically associated with alkyl-substituted BTBT derivatives. Among the three, compound **1** undergoes a direct transition from a crystalline structure to an isotropic state at a relatively low temperature. In contrast, compounds **2** and **3** demonstrate a low-temperature crystal-to-crystal transition, associated with molecular rearrangement within the unit cells. At higher temperatures, the Cr2 phase transforms into a layered SmB structure, likely involving a significant reorganization of molecular conformations and their relative positions.

The SmB phase persists over a wide temperature range, eventually transitioning to a SmA phase in compound **3** or an isotropic state in compound **2**. It is possible that the narrow temperature window of the SmA phase in compound **2** was not detected in the experiment. These findings underscore the substantial influence of side-chain attachment positions on the BTBT core, profoundly affecting both the molecular packing and phase behavior of these compounds.

Semiconducting properties of compounds **1**–**3** were investigated in OFETs prepared by different techniques. It was found that mobility increases in the row of compounds **2** < **1** < **3**, while for each compound the best conditions were different. It could be explained by different solubility and phase behavior of each compound. Compound **3**, having the most linear structure, the lowest solubility, and a higher degree of ordering (estimated as a sum of the entropy of phase transitions when heating from room temperature to the isotropic melt), possesses the best electrical characteristics in solution-processible OFETs. In spite of lower charge carrier mobility, compounds **1** and **2** contain active protons at position 5 of thiophene rings, which opens up the possibility to use them as building blocks for further synthesis of conjugated polymers, which could have different phase behavior and semiconducting properties.

## Figures and Tables

**Table 1:** Photophysical properties of the compounds investigated.

Feature	Compound			
	1	2	3	BTBT
$\lambda_{abs}^{\max}$ * (nm)	340	385	389	331
$\varepsilon_{abs}^{\max}$ * (×10^4^, M^−1^ cm^−1^)	4.57	5.04	5.22	1.46
$\lambda_{fluor}^{\max}$(nm)	393; 411; 438	398; 421; 446	402; 425; 450; 485	337; 352; 368
0-0 transition (cm^−1^)	26,500	25,400	25,200	30,000
Stokes shift (cm^−1^)	2200	640	630	540
QY (%)	40	63	73	3
τ (ns)	0.52	0.78	0.82	0.19
k_r_ (ns^−1^)	0.77	0.81	0.89	0.16
k_nr_ (ns^−1^)	1.2	0.5	0.3	5.1

Notes. $\lambda_{abs}^{\max}$—absorption maximum, $\varepsilon_{abs}^{\max}$—molar extinction coefficient, $\lambda_{fluor}^{\max}$—fluorescent maximum, QY—quantum yield, τ—luminescence lifetime, k_r_—radiative constant, k_nr_—nonradiative constant. * Major maxima.

**Table 2:** Frontier molecular orbital energies, oxidation/reduction potentials, and energy gaps obtained by theoretical calculations and extracted from CV and absorption spectra measurements.

Compound	$E_{HOMO/LUMO}^{DFT}$ (eV)	$E_{ox\;vs(Fc/{Fc}^{+})}^{onset}/E_{red\;vs(Fc/{Fc}^{+})}^{onset}$ (V)	$E_{HOMO/LUMO}^{CV}$ (eV)	$E_{gap}^{DFT}$ (eV)	$E_{gap}^{CV}$ (eV)	$E_{gap}^{opt}(THF)$ (eV)	$E_{gap}^{opt}(film)$ (eV)
**1**	−5.79/−1.74	0.79/−2.45	−5.59/−2.35	4.05	3.24	3.05	2.96
**2**	−5.61/−1.82	0.81/−2.45	−5.61/−2.35	3.79	3.26	2.97	2.87
**3**	−5.51/−1.77	0.97/−2.37	−5.77/−2.43	3.74	3.34	2.95	2.83

Notes. $E_{HOMO/LUMO}^{DFT}$—HOMO and LUMO energy levels estimated by DFT calculations, $E_{ox\;vs(Fc/{Fc}^{+})}^{onset}/E_{red\;vs(Fc/{Fc}^{+})}^{onset}$—oxidation/reduction potentials obtained from CV measurements, $E_{HOMO/LUMO}^{CV}$—HOMO and LUMO energy levels obtained from CV measurements, $E_{gap}^{DFT}$—energy gap estimated by DFT calculations, $E_{gap}^{CV}$—energy gap obtained from CV measurements, $E_{gap}^{opt}$ (THF)—energy gap obtained from optical measurements in THF solutions, $E_{gap}^{opt}$ (film)—energy gap obtained from optical measurements in polycrystalline thin films.

**Table 3:** Data on phase transitions obtained from the first heating DSC scans.

Compound	Temperature (^°^C)	ΔH (kJmol^−1^)	ΔS (J mol^−1^K^−1^)
**1**	81	7.2	20.3
	96	14.5	39.4
**2**	91	1.42	3.9
	103	0.66	1.8
	158	1.93	4.5
	250	13.51	25.8
	258	9.89	18.6
**3**	122	18.68	47.3
	285	14.03	25.1
	333	12.64	20.9

Notes: ΔH—enthalpy of phase transition, ΔS—enthalpy of phase transition.

**Table 4:** Thermal transitions of samples investigated, as determined by variable-temperature synchrotron SAXS/WAXS analysis.

Compound	Transitions
**1**	Cr, 93 °C; Isotropic
**2**	Cr1, 97–110 °C; Cr2, 157 °C; SmB, 273 °C; (SmA); Isotropic
**3**	Cr1, 60 °C; Cr2, 122 °C; SmB, 293 °C; SmA

Notes: Cr, Cr1, Cr2—different crystal phases, SmB—ordered smectic B mesophase, SmA—disordered smectic A mesophase.

**Table 5:** Characteristics of the best OFET devices from compounds **1**–**3** and reference material **C8-BTBT** for different methods.

Compound	Preparation Method	$\mu_{sat}$, cm^2^V^−1^s^−1^	*Vth, V*	*I_on/off_*	$\mu_{eff}$, cm^2^V^−1^s^−1^
**1**	SC/TC	0.24 ± 0.01	−17.2 ± 0.5	9.00 × 10^6^	0.076 ± 0.002
**C8-BTBT**	SC/TC	(4.3 ± 0.1) × 10^−3^	8.6 ± 1.1	5.20 × 10^5^	(2.3 ± 0.2) × 10^−3^
**2**	DB/TC	(2.8 ± 0.1) × 10^−4^	0.2 ± 1.3	2.90 × 10^4^	(2.5 ± 0.2) × 10^−4^
**3**	DB/TC	0.045 ± 0.001	−14.0 ± 0.5	6.90 × 10^5^	0.019 ± 0.001
**C8-BTBT**	DB/TC	(7.1 ± 0.2) × 10^−5^	−12.5 ± 0.8	3.00 × 10^5^	(3.2 ± 0.2) × 10^−5^
**3**	DB/BC	(6.4 ± 0.1) × 10^−3^	2.4 ± 0.5	4.30 × 10^5^	(6.9 ± 0.3) × 10^−3^
**C8-BTBT**	DB/BC	1.50 ± 0.04	4.4 ± 0.8	3.10 × 10^9^	1.70 ± 0.10
**3**	DC/BC	0.46 ± 0.03	14.0 ± 2.3	6.80 × 10^7^	1.10 ± 0.14
**C8-BTBT**	DC/BC	(3.1 ± 0.1) × 10^−4^	9.9 ± 0.2	5.60 × 10^4^	(4.8 ± 0.1) × 10^−4^

Notes: SC—spin coating, DB—doctor blade, DC—drop casting, TC—top contact, BC—bottom contact, *µ_sat_*—the maximum mobility in the saturated regime, Vth—the threshold voltage, *I_on_/I_off_*—the on/off ratio, *µ_eff_*—effective mobility calculated according to [42].

## Data Availability

The original contributions presented in this study are included in the article/Supplementary Materials. Further inquiries can be directed to the corresponding author.

## Supplementary Materials

The following supporting information can be downloaded at: https://www.mdpi.com/article/10.3390/ma18040743/s1. Figures S1–S6: ^1^H and ^13^C NMR spectra of compounds **1**–**3**; Figures S7–S9: Experimental and simulated HRMS of compounds **1**–**3**; Figures S10–S12: GPC curves (a) and corresponding absorption spectra (b) of the starting materials and target of compounds **1**–**3** after purification; Figure S13: Visualization of frontier molecular orbitals of compounds **1**–**3**; Figure S14: Absorption, fluorescence, and excitation spectra of thin films as compared to the spectra of diluted solutions of compounds **1**–**3**; Figure S15: TGA curves of compounds **1**–**3**; Figure S16: POM microphotos of compounds **1**, **2**, and **3** in cross-polarizers; Table S1: Results of TD DFT (PBE0) calculations; Tables S2–S9: Containing examples of preparation conditions and electrical characteristics of the OFETs measured.

## Abbreviations

The following abbreviations are used in this manuscript:

BC	bottom contact
BTBT	[1]benzothiopheno[3,2-b][1]benzothiophene
CV	cyclic voltammetry
DB	doctor blade
DC	drop casting
DCB	dichlorobenzene
DSC	differential scanning calorimetry
EGOFET	electrolyte-gated organic field-effect transistor
ESRF	European Synchrotron Radiation Facility
GPC	gel permeation chromatography
HOMO	highest occupied molecular orbital
LUMO	lowest unoccupied molecular orbital
NMR	nuclear magnetic resonance
OFET	organic field-effect transistor
OSC	organic semiconductor
PFBT	pentafluorobenzenethiol
POM	polarized optical microscopy
SAXS	small-angle X-ray scattering
SC	spin coating
SmA	smectic A mesophase
SmB	smectic B mesophase
TC	top contact
TD DFT	Time-Dependent Density Functional Theory
TGA	thermogravimetric analysis
QY	quantum yield
WAXS	wide-angle X-ray scattering
