# Supplementary material for: Three Isomeric Dioctyl Derivatives of 2,7-Dithienyl[1]benzo-thieno[3,2-b][1]benzothiophene: Synthesis, Optical, Thermal, and Semiconductor Properties
Source: Materials (Basel). 2025 Feb 7;18(4):743. doi: 10.3390/ma18040743 (PMC11857614; doi:10.3390/ma18040743)
Supplement: Supplementary file 1 [file materials-18-00743-s001.zip › materials-3426915-supplementary/Materials_ESI_revised_corrected.pdf]

Electronic supplementary materials

Three isomers of dioctyl derivatives of 2,7-dithienyl[1]benzothieno[3,2-b][1]benzothiophene: synthesis, optical, thermal and semiconductor properties

Lev L. Levkov[1], Nikolay M. Surin[1], Oleg V. Borshchev [1], Yaroslava O. Titova[1], Nikita O. Dubinets[1], Evgeniya A. Svidchenko[1], Polina S. Shaposhnik[1], Askold A. Trul[1], Akmal Z. Umarov[3], Denis V. Anokhin [2,3], Martin Rosenthal [5], Dimitri A. Ivanov[2,3,4], Victor V. Ivanov[6], Sergey A. Ponomarenko [1,2] *

[1] Enikolopov Institute of Synthetic Polymeric Materials Russian Academy of Sciences, Profsoyuznaya Str. 70, Moscow 117393, Russia
[2] Faculty of Chemistry, Lomonosov Moscow State University, Leninskie Gory 1/73, Moscow 119991, Russia
[3] Federal Research Center of Problems of Chemical Physics and Medicinal Chemistry RAS, Chernogolovka, 142432 Moscow region, Russia
[4] Institut de Sciences des Matériaux de Mulhouse-IS2M, CNRS UMR 7361, Jean Starcky, 15, F-68057 Mulhouse, France
[5] Faculty of Chemistry, KU Leuven, Celestijnenlaan 200F, Box 2404, B-3001 Leuven, Belgium
[6] Moscow Center for Advanced Studies, Kulakova str. 20, 123592, Moscow, Russia
* Correspondence: ponomarenko@ispm.ru

Contents:

1. **NMR spectra** **S2**
2. **HRMS spectra** **S5**
3. **GPC curves** **S6**
4. **DFT calculations** **S8**
5. **Optical properties of thin films** **S9**
6. **TGA curves** **S10**
7. **POM images** **S11**

1. NMR spectra

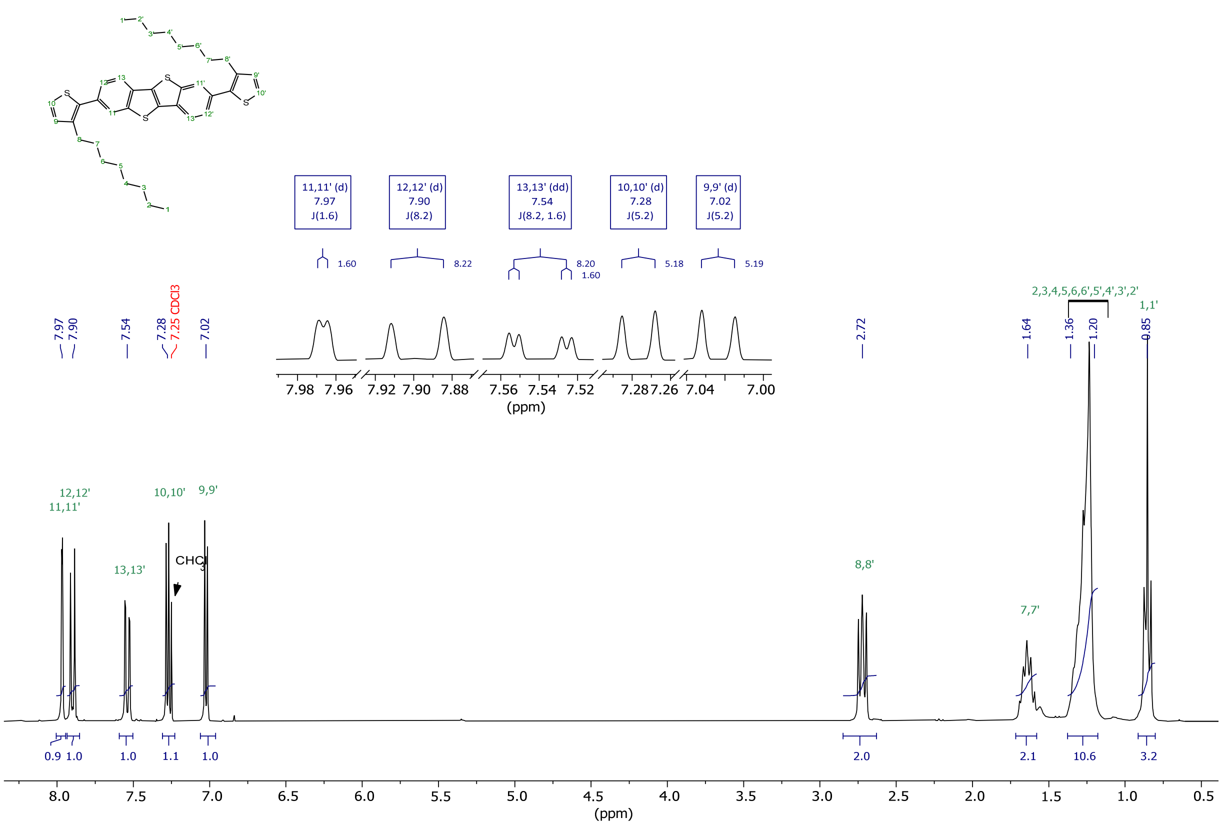

Figure S1. ^{1}H NMR spectrum of compound **1** in $CDCl_3$.

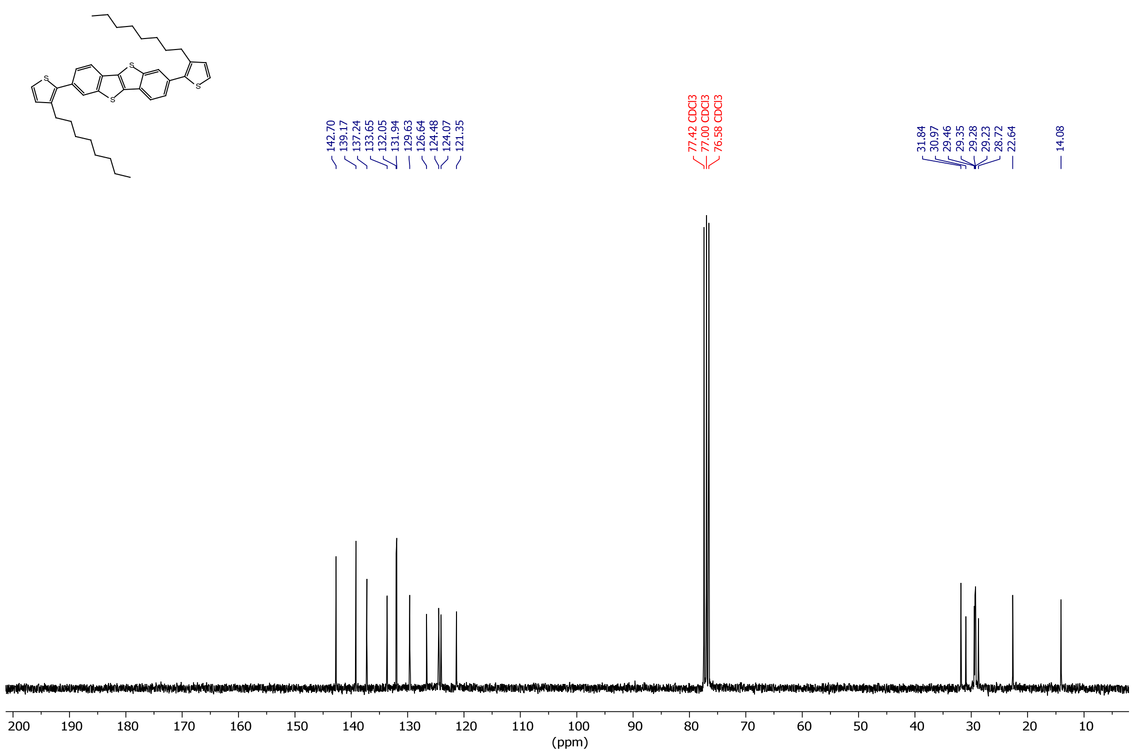

Figure S2. ^{13}C NMR spectrum of compound **1** in $CDCl_3$. Note: signal at 28.72 ppm correspond to a residual amount of cyclohexane.

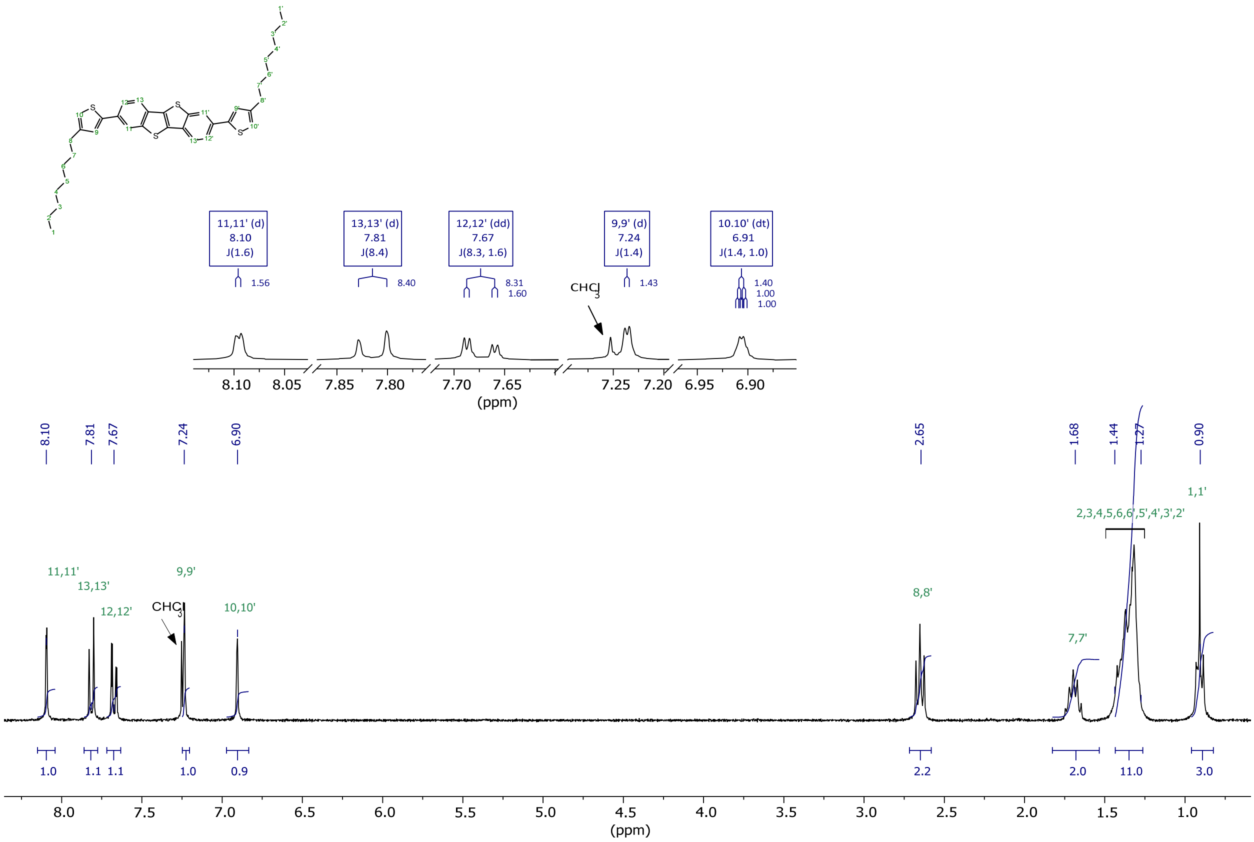

Figure S3. ^{1}H NMR spectrum of compound **2**.

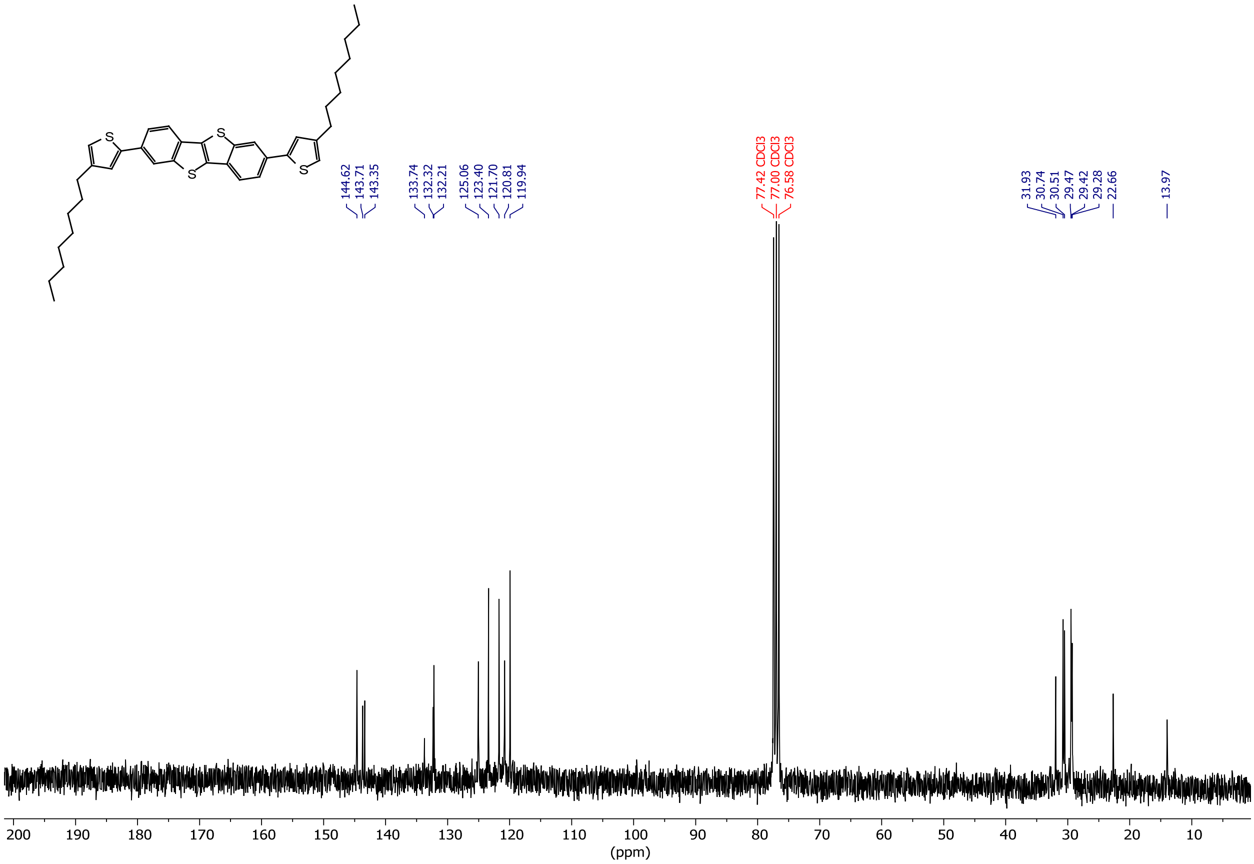

Figure S4. ^{13}C NMR spectrum of compound **2**.

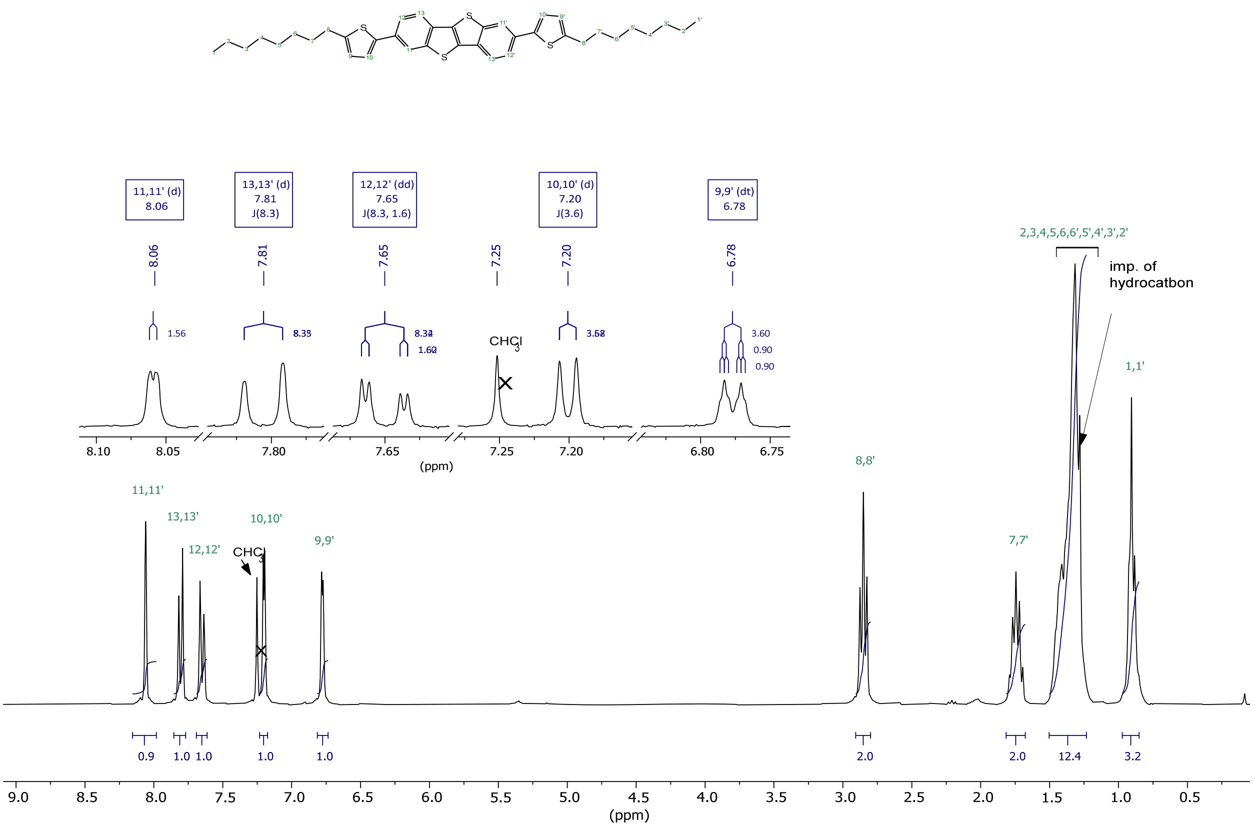

Figure S5. ^{1}H NMR spectrum of compound **3.**

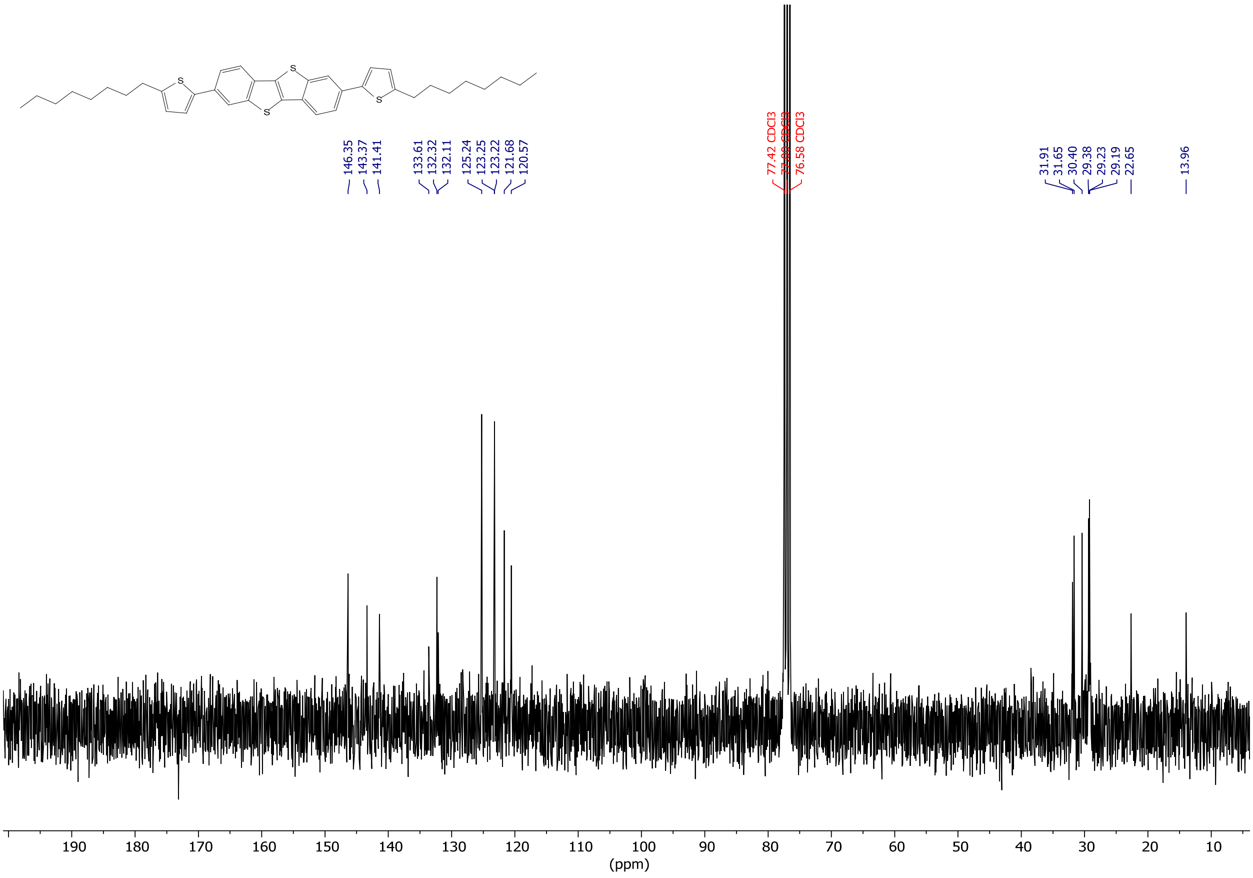

Figure S6. ^{13}C NMR spectrum of compound **3.**

2. HRMS spectra

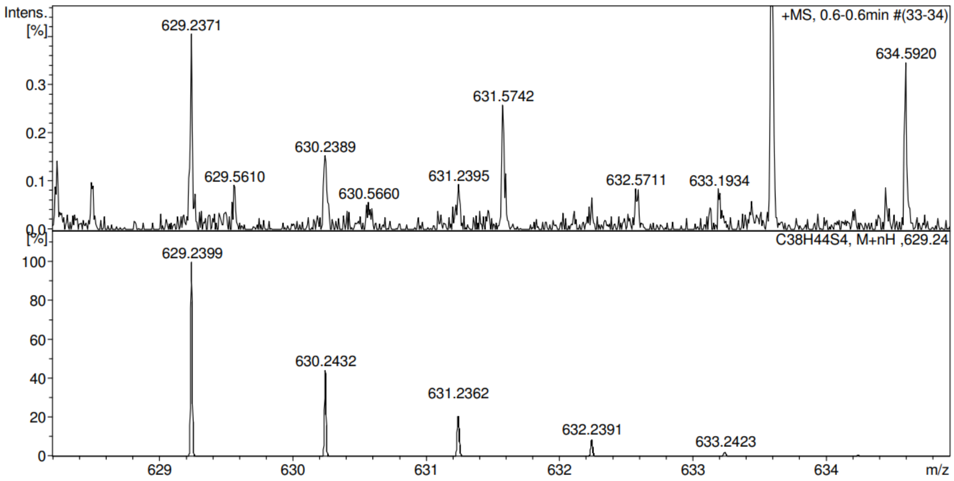

Figure S7. Experimental and simulated HRMS spectra of compound **1**.

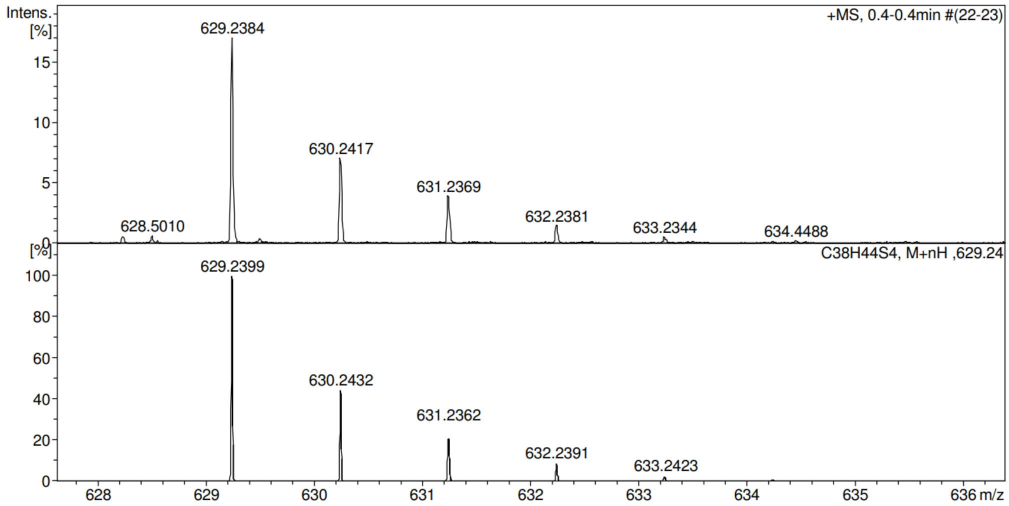

Figure S8. Experimental and simulated HRMS spectra of compound **2**.

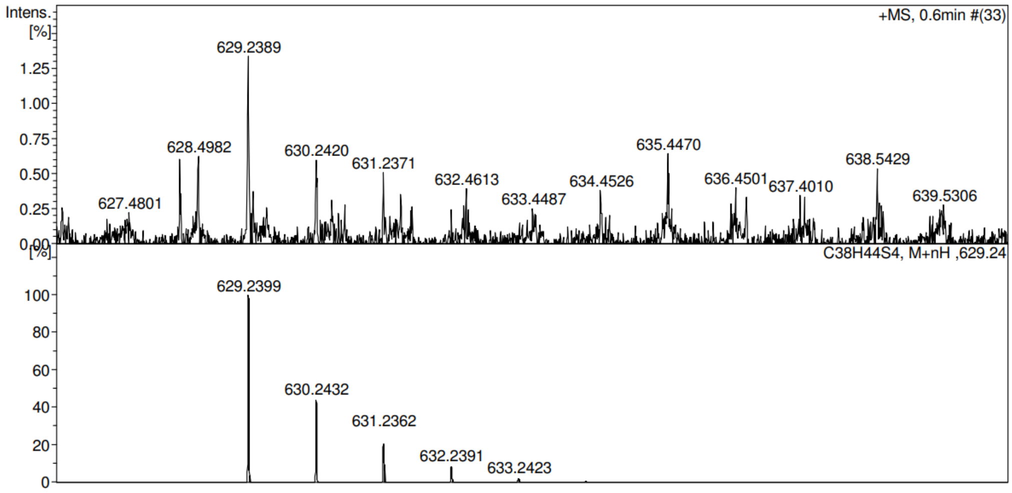

Figure S9. Experimental and simulated HRMS spectra of compound **3**.

3. GPC curves

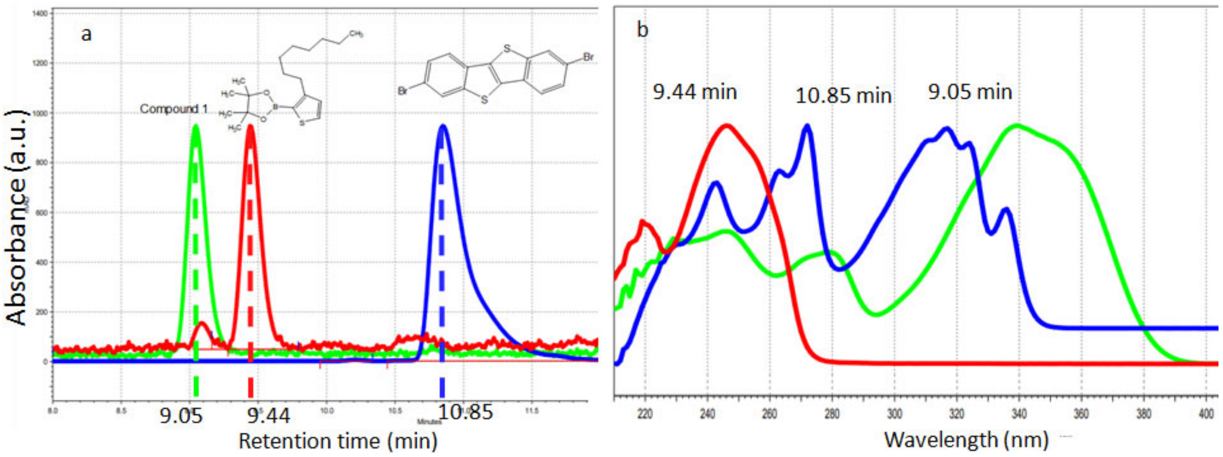

Figure S10. GPC curves (a) and corresponding absorption spectra (b) of the starting materials and target compound **1** after purification.

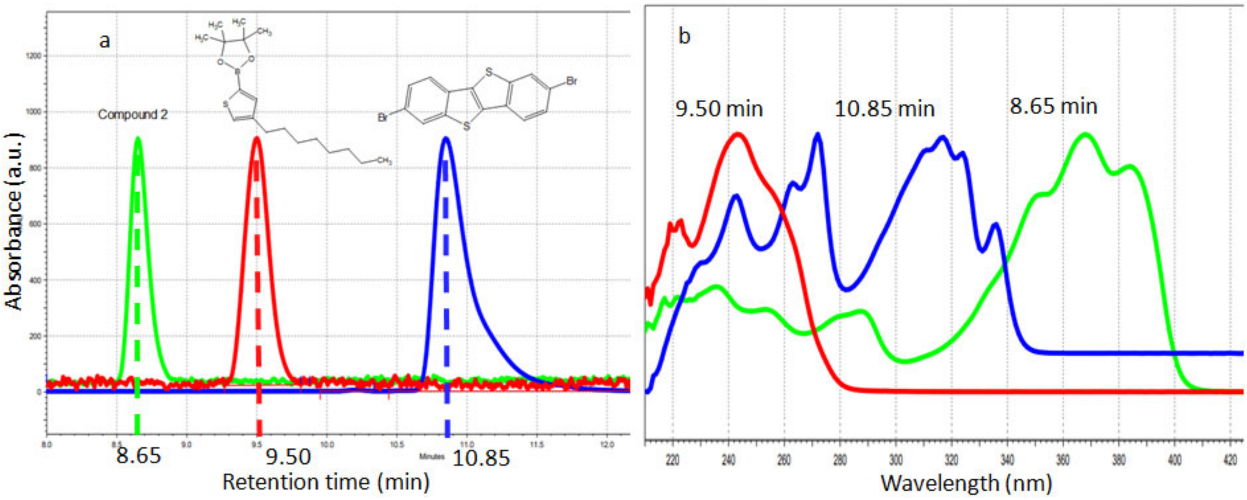

Figure S11. GPC curves (a) and corresponding absorption spectra (b) of the starting materials and target compound **2** after purification

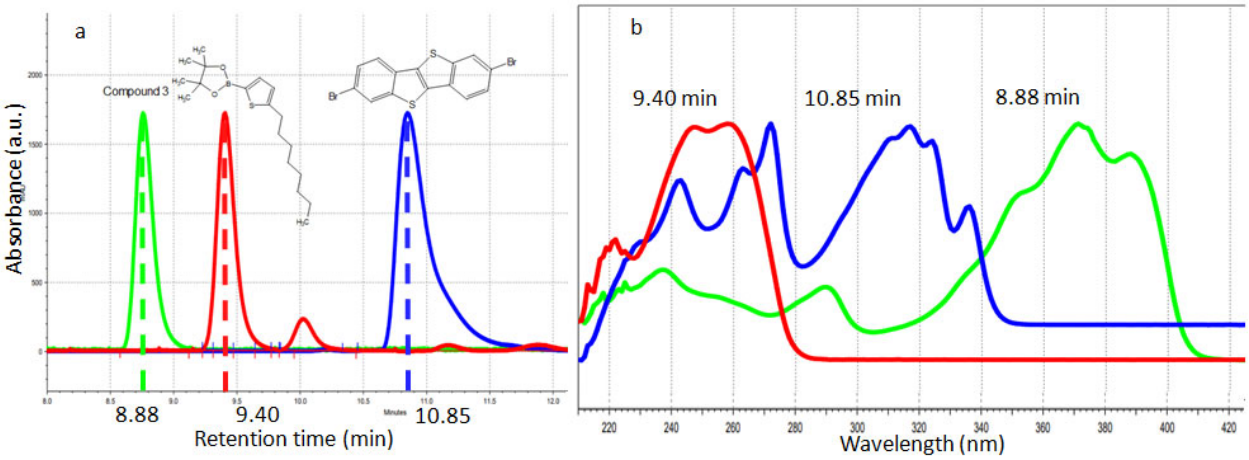

Figure S12. GPC curves (a) and corresponding absorption spectra (b) of the starting materials and target compound **3** after purification

4. DFT calculations

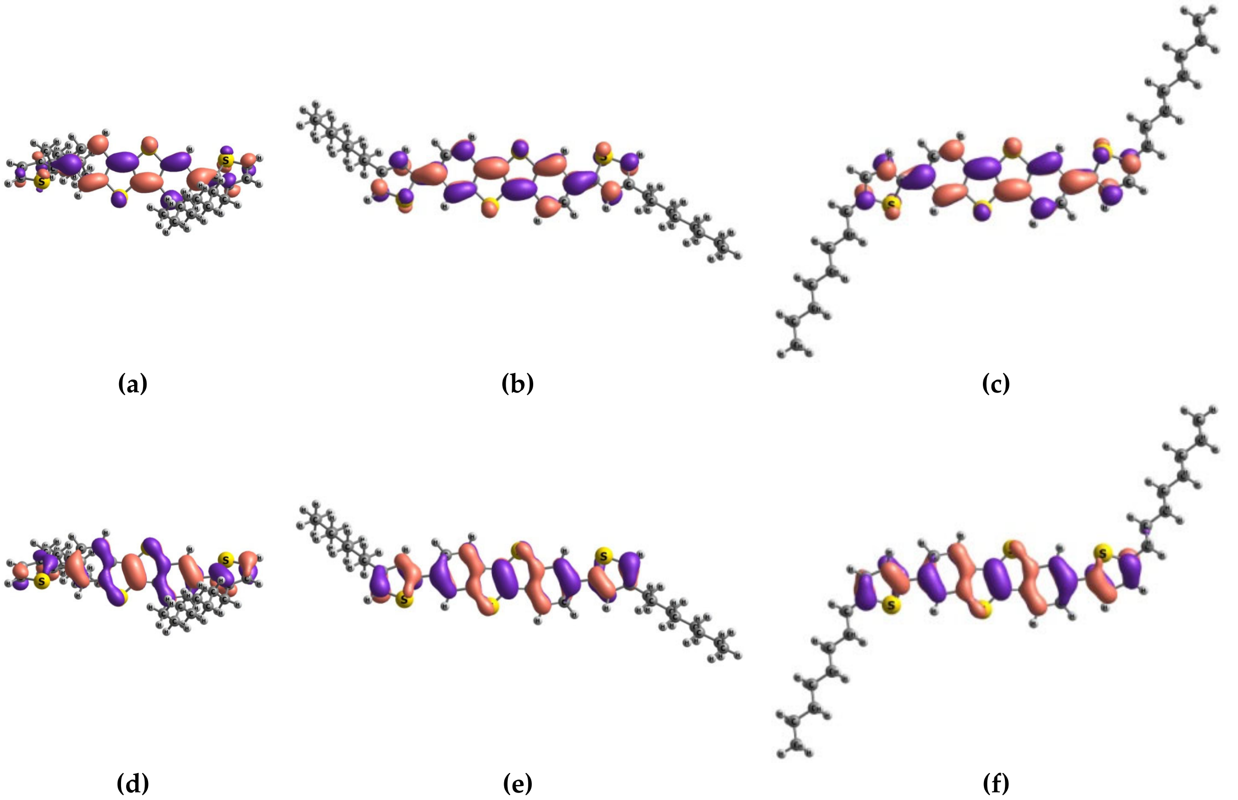

Figure S13. Visualization of frontier molecular orbitals of compounds **1** (a,d), **2** (b,e) and **3**(c,f): LUMO (a, b, c) and HOMO (d, e, f).

Table. S1. Results of TD DFT (PBE0) calculations

Feature		Compound			
	BTBT	BTBT-T	1	2	3
HOMO, eV	-6.00	-5.70	-5.79	-5.61	-5.51
LUMO, eV	-1.45	-1.90	-1.74	-1.82	-1.77
Eg, eV	4.56	3.80	4.05	3.79	3.74
Transition, nm/fosc	317.6/0.136<br>290.3/0.328<br>277.4/0<br>262.8/0<br>259.7/0<br>255.9/0.301<br>23.8/0<br>234.2/0	376.2/1.261<br>333.2/0.226<br>305.5/0<br>301.2/0<br>297.4/0<br>281.4/0.245<br>269.5/0<br>268.9/0<br>264/0<br>258.9/0.035	356.6/1.031<br>322.9/0.183<br>301.6/0<br>296.4/0.002<br>280.3/0.085<br>279.2/0.121<br>268.3/0.096<br>266.6/0.001<br>265.1/0.002<br>263.3/0.001	378.7/1.457<br>332.4/0.199<br>308.5/0<br>303.8/0.001<br>297.4/0.001<br>283.2/0.239<br>271.1/0.069<br>270.7/0.001<br>268.5/0.001<br>265.9/0.001	385/1.581<br>333.3/0.174<br>314.3/0<br>30.7/0<br>30.2/0<br>285.9/0.275<br>272.5/0<br>269.6/0<br>263.5/0.001<br>262.6/0.09
Dihedral angle, °	–	-24.07	53.732	28.74	-25.87
E (S_0-S_1), cm^{-1}	–	26600	28000	26400	26000
E (T_1), cm^{-1}	–	16000	16200	15900	15600
E (S_0-S_1) - E (T_1), cm^{-1}	–	10600	11800	10500	10400

5. Optical properties of thin films

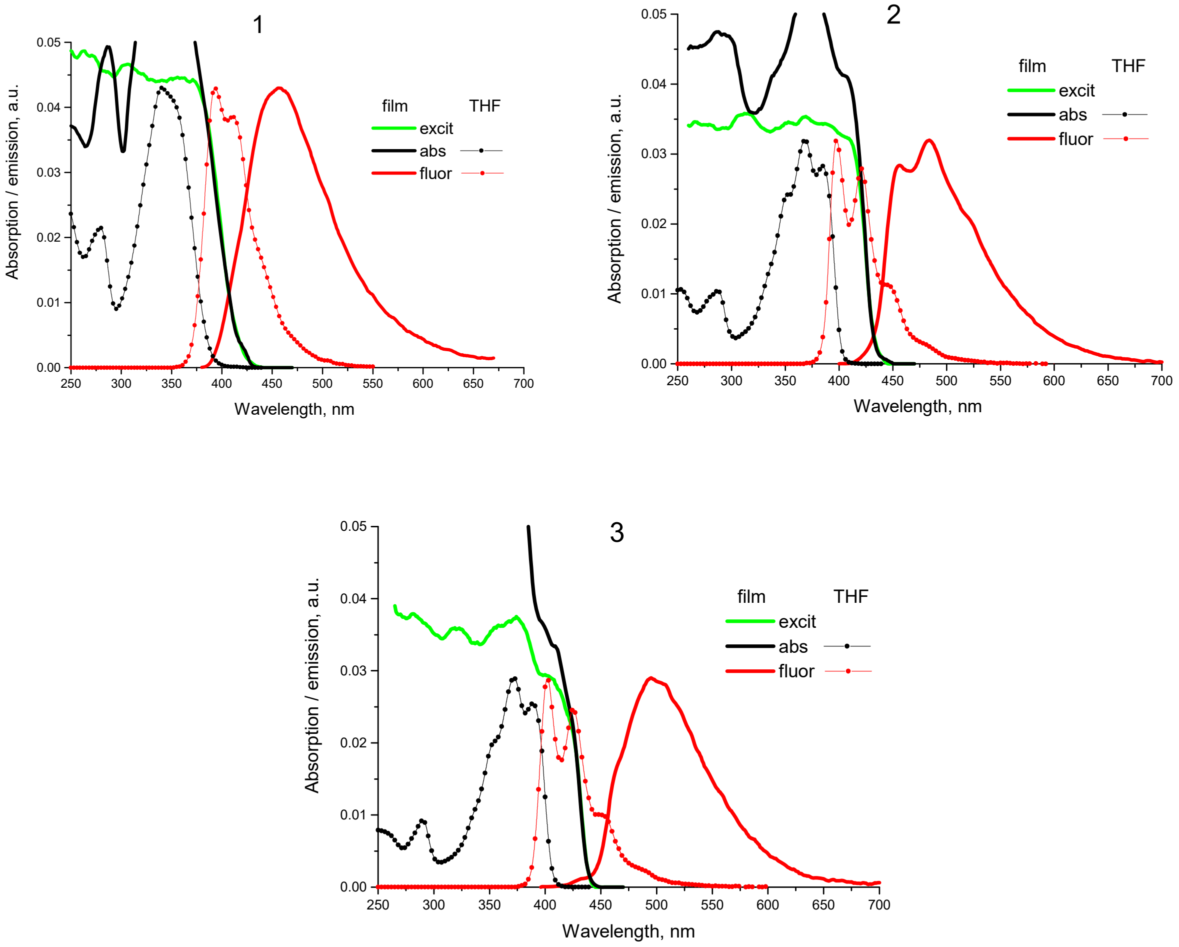

Figure S14. Absorption (**black**), fluorescence (**red**) and excitation (**green**) spectra of thin films (thick solid lines) as compared to the spectra of diluted solutions (thin lines with dots) of compounds 1 – 3. For the thin film measurements, the excitation wavelength was 350 (1), 370 (2) and 368 (3) nm, while excitation spectra were recorded at registration on 460 (1), 490 (2), 500 (3) nm wavelengths for compounds **1**, **2** and **3**, respectively.

6. TGA curves

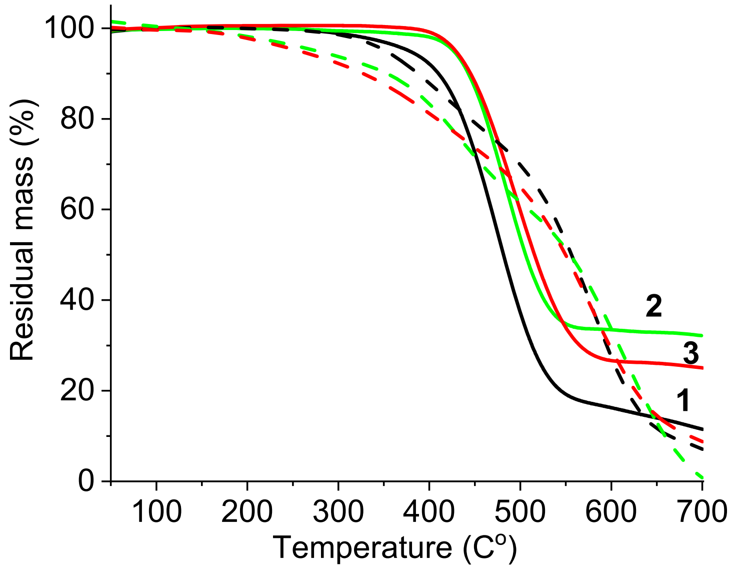

Figure S15. TGA curves of compounds **1**, **2** and **3**: solid lines in argon atmosphere. dashed line - in the air.

7. POM images

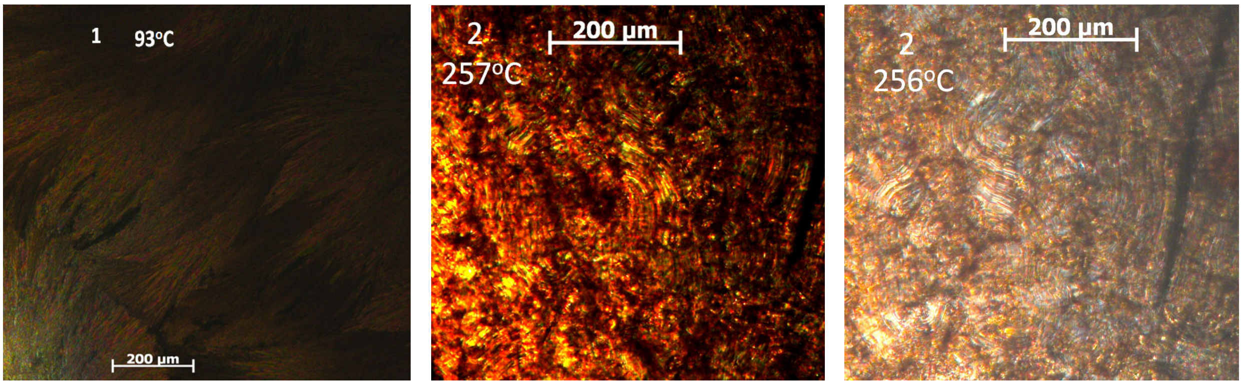

(a) (b) (c)

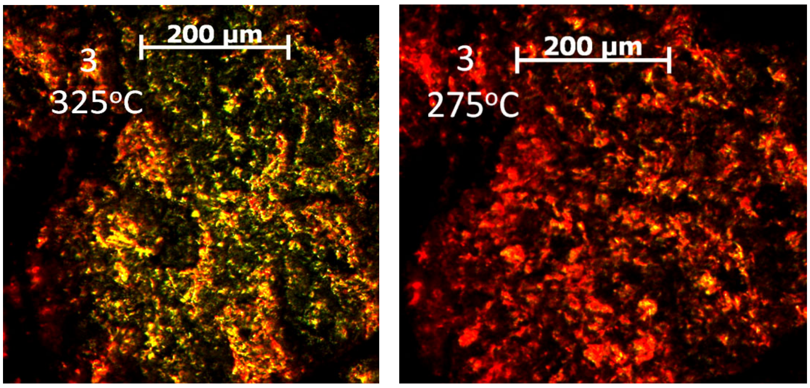

(d) (e)

Figure S16. POM microphotos of compounds **1**, **2** and **3** in cross-polarizers at different temperatures after cooling from isotropic melt: compound **1** at 93 ºC (a), compound **2** at 257 ºC (b) and 256 ºC (c), compound **3** at 325 ºC (d) and 275 ºC (e).
